# Molecular Characterization and Sex Distribution of Chemosensory Receptor Gene Family Based on Transcriptome Analysis of *Scaeva pyrastri*

**DOI:** 10.1371/journal.pone.0155323

**Published:** 2016-05-12

**Authors:** Xiao-Ming Li, Xiu-Yun Zhu, Peng He, Lu Xu, Liang Sun, Li Chen, Zhi-Qiang Wang, Dao-Gui Deng, Ya-Nan Zhang

**Affiliations:** 1 College of Life Sciences, Huaibei Normal University, Huaibei, China; 2 State Key Laboratory Breeding Base of Green Pesticide and Agricultural Bioengineering, Key Laboratory of Green Pesticide and Agricultural Bioengineering, Ministry of Education, Guizhou University, Guiyang, China; 3 Institute of Plant Protection, Jiangsu Academy of Agricultural Sciences, Nanjing, China; 4 Key Laboratory of Tea Biology and Resources Utilization, Ministry of Agriculture, Tea Research Institute, Chinese Academy of Agricultural Sciences, Hangzhou, China; USDA-ARS, UNITED STATES

## Abstract

Chemosensory receptors play key roles in insect behavior. Thus, genes encoding these receptors have great potential for use in integrated pest management. The hover fly *Scaeva pyrastri* (L.) is an important pollinating insect and a natural enemy of aphids, mainly distributed in the Palearctic and Nearctic regions. However, a systematic identification of their chemosensory receptor genes in the antennae has not been reported. In the present study, we assembled the antennal transcriptome of *S*. *pyrastri* by using Illumina sequencing technology. Analysis of the transcriptome data identified 60 candidate chemosensory genes, including 38 for odorant receptors (*ORs*), 16 for ionotropic receptors (*IRs*), and 6 for gustatory receptors (*GRs*). The numbers are similar to those of other Diptera species, suggesting that we were able to successfully identify *S*. *pyrastri* chemosensory genes. We analyzed the expression patterns of all genes by using reverse transcriptase PCR (RT-PCR), and found that some genes exhibited sex-biased or sex-specific expression. These candidate chemosensory genes and their tissue expression profiles provide information for further studies aimed at fully understanding the molecular basis behind chemoreception-related behaviors in *S*. *pyrastri*.

## Introduction

An accurate and complex olfactory system helps insects find resources (e.g., suitable hosts, predators, oviposition sites, mates) [1]. Previous studies have shown that insects tend to use their antenna—an efficient olfactory organ—to detect chemical signals from the external environment [2, 3]. The insect olfactory system involves several molecular components, including odorant binding proteins (OBPs) [4–6], chemosensory proteins (CSPs) [7, 8], and sensory neuron membrane proteins (SNMPs) [9, 10]. Additionally, 3 major chemosensory receptor families are involved: olfactory receptors (ORs) [1, 11, 12], gustatory receptors (GRs) [13–15], and ionotropic receptors (IRs) [4, 16, 17]. These receptors are located on the dendrites of neurons in antenna chemosensilla and other chemosensory tissues.

To explore the mechanisms underlying insect olfaction, the identification, sex distribution, and functional analyses of candidate chemosensory receptor genes are important initial steps. Compared with older gene cloning techniques such as rapid amplification of cDNA ends (RACE) and expressed sequence tag (EST) library construction [18–22], next-generation sequencing techniques such as RNA sequencing (RNA-seq) are now considered more efficient in generating data, less time-consuming, and more cost-effective. These recent technological advancements have allowed the large-scale identification of chemosensory genes from Diptera insects whose genomes are not yet sequenced, as is the case of *Calliphora stygia* [23], *Bactrocera dorsalis* [24], *Mayetiola destructor* [25], and the natural enemy insect *Microplitis mediator* [26]. However, their exact functions are largely unknown, as these genes were mainly identified based on sequence similarity to reported genes. Their expression profiles, particularly those varying according to sex, and phylogenetic analyses could provide important information on the functions of chemosensory receptor genes [25, 27–30].

*Scaeva pyrastri* (L.) (Diptera: Syrphidae) is a pollinating and natural enemy insect found worldwide, although mainly distributed in the Palearctic and Nearctic [31]. Adults are flower-visiting and larvae prey on aphids, a major agricultural pest [32]. Previous studies have shown that some chemical cues (plant volatiles and the residues or secretions of aphids) play a key role in mediating many aspects of *S*. *pyrastri* behavior, such as parasitism and oviposition [33–35], but the specific molecular mechanisms of their chemosensory-guided behaviors are currently unknown. In the present study, we performed a transcriptome analysis based on adult *S*. *pyrastri* antennae, and identified 60 candidate chemosensory receptor genes comprising 38 *ORs*, 6 *GRs*, and 16 *IRs*. We further conducted a comprehensive analysis of their phylogeny and sex distribution and the results clearly demonstrated that some genes exhibit sex-biased or -specific expression. Thus, our data contribute to the overall understanding of chemoreception-based behavioral mechanisms in *S*. *pyrastri*.

## Materials and Methods

### Ethics statement

*S*. *pyrastri* were collected in April 2015 from a *Brassica campestris* field in the Pollution-Free Planting Base of Huaibei City, Anhui Province, China. The field studies did not involve endangered or protected species, and no specific permissions were required for these research activities in these locations.

### Insect rearing and collection

Adult *S*. *pyrastri* were separated into females and males, and reared on aphids. The rearing conditions were 25 ± 1°C, 12:12 light:dark photoperiod, and 70 ± 10% relative humidity. For transcriptome sequencing, the antennae of 600 adults (300 males and 300 females) were collected. For the tissue expression study, 150–200 female antennae (FA), 150–200 male antennae (MA), and 12–15 whole insect bodies without antennae (Bo) were also collected. All samples were immediately frozen in liquid nitrogen and stored at -80°C until use.

### cDNA library construction, clustering, and sequencing

As previously described in detail [36–38], total RNA was extracted using TRIzol reagent (Invitrogen, Carlsbad, CA, USA). Construction of the cDNA library and Illumina sequencing were performed at Novogene Bioinformatics Technology Co., Ltd., Beijing, China. The mRNA was purified from 3 μg of total RNA using oligo (dT) magnetic beads and fragmented into short sequences in the presence of divalent cations at 94°C for 5 min. First-strand cDNA was then generated using random hexamer-primed reverse transcription, followed by second-strand-cDNA synthesis using RNaseH and DNA polymerase I. After adaptor end-repair and ligation, cDNA was amplified via PCR and purified using the QIAquick PCR Purification Kit to create a cDNA library. Library quality was assessed on an Agilent Bioanalyzer 2100 system. Clustering of the index-coded samples was performed on a cBot Cluster Generation System using a TruSeq PE Cluster Kit v3-cBot-HS (Illumina), following the manufacturer’s protocol. After cluster generation, library preparations were sequenced on an Illumina Hiseq^™^ 2500 platform and paired-end reads were obtained.

### *De novo* assembly of short reads and gene annotation

Raw reads were cleaned following the methods described in our previous studies [36–38], by removing reads with low-quality and/or containing adapters or poly-N tails. Transcriptome *de novo* assembly was performed based on clean short reads using the program Trinity (r20140413p1) [39, 40] with its default parameters. The Basic Local Alignment Search Tool (BLASTX) was used to search for sequence homology between unigenes > 150 bp resulting from the assembly and sequences deposited in the National Center for Biotechnology Information (NCBI) Non-redundant (Nr), Swiss-Prot, Kyoto Encyclopedia for Genes and Genomes (KEGG), and Clusters of Orthologous Groups (COG) databases (e-value < 10^−5^ for all databases). Proteins with the highest sequence similarity were retrieved, along with their functional annotations. We then used Blast2GO (e-value < 10^−6^) [41] for gene ontology (GO) annotation and functional classification of the unigenes.

### Sequence analyses

The open reading frames (ORFs) of chemosensory genes were predicted using ORF Finder (http://www.ncbi.nlm.nih.gov/gorf/gorf.html). Similarity searches were performed using the NCBI-BLAST network server (http://blast.ncbi.nlm.nih.gov/). The transmembrane domains of *S*. *pyrastri* ORs, IRs, and GRs (SpyrORs, SpyrIRs, and SpyrGRs, respectively) were predicted with the TMHMM Server Version 2.0 (http://www.cbs.dtu.dk/services/TMHMM).

### Phylogenetic analyses

The phylogenetic trees of SpyrORs, SpyrGRs, and SpyrIRs were reconstructed based on the sequences obtained here and on the amino acid sequences of ORs, GRs, and IRs reported for other insects. The OR data set contained 38 sequences from *S*. *pyrastri*, plus 204 combined from *Drosophila melanogaster* [42], *C*. *stygia* [23], *Acyrthosiphon pisum* [43, 44], and *A*. *gossypii* [45]. The GR data set contained 6 sequences from *S*. *pyrastri*, plus 280 combined from *D*. *melanogaster* [42], *Anopheles gambiae* [46], and *Bombyx mori* [47]. The IR data set contained 16 sequences from *S*. *pyrastri*, plus 154 combined from *D*. *melanogaster* [48], *A*. *gossypii* [45], *Musca domestica*, *A*. *gambiae*, and *C*. *stygia* [23]. The amino acid sequences of *S*. *pyrastri* genes used for phylogenetic tree construction are listed in S1 Table. Amino acid sequences were aligned using ClustalX 1.83 [49] and the phylogenetic trees were constructed in PhyML [50], based on the LG substitution model [51] with Nearest Neighbor Interchange(NNI); branch support was estimated with a Bayesian-like transformation of aLRT (aBayes). Dendrograms were created and colored in FigTree (http://tree.bio.ed.ac.uk/software/figtree/).

### RNA isolation, cDNA synthesis, and reverse transcription-PCR analysis

As previously described in detail [36–38], total RNA was extracted with the SV 96 Total RNA Isolation System (Promega, Madison, WI, USA) following the manufacturer’s protocol, and including a DNaseI digestion to avoid genomic DNA contamination. RNA quality was verified with a NanoDrop^™^ 2000 (Thermo Fisher Scientific, USA). Single-stranded cDNA templates were synthesized using 1 μg of total RNA from both body and antennae tissue samples and the PrimeScript^™^ RT Master Mix (TaKaRa, Dalian, China). Gene-specific primers across the ORFs of predicted chemosensory genes were designed using Primer Premier 5.0 (PREMIER Biosoft International, CA, USA), and their sequences are listed in S2 Table. Reverse transcription (RT)-PCR (including negative controls with no cDNA template) profile was as follows: initial denaturation at 94°C for 4 min; 35–40 cycles at 94°C for 30 s, 60°C for 30 s, and 72°C for 40 s; and final incubation at 72°C for 10 min. Cycle number was reduced to 30 in the reference gene amplification. The reaction volume was 25 μL, containing 12.5 μL Premix Taq^™^ (TaKaRa Taq^™^ Version 2.0; TaKaRa, Dalian, China), 0.4 μM each primer, 1 μL sample cDNA (15 ng/μL), and 9.5 μL sterilized H_2_O. PCR products were analyzed via electrophoresis on 1.5% w/v agarose gels in TAE buffer (40 mmol/L Tris-acetate, 2 mmol/L Na_2_EDTA·H_2_O), and the resulting bands were visualized using SYBR Green I (Tiandz, Beijing, China). The gene encoding *S*. *pyrastri* glyceraldehyde-3-phosphate dehydrogenase (*SpyrGAPDH*) was used as reference to check the integrity of the cDNA template, and a reagents mix without cDNA template was used as the negative control (NC). Two independent biological replications were performed for each RT-PCR amplification, and each biological replication was repeated at least twice. The expected products of randomly selected genes were sequenced to confirm they corresponded to the originally identified sequence.

## Results

### Transcriptome sequencing and sequence assembly

**Fig 1:**
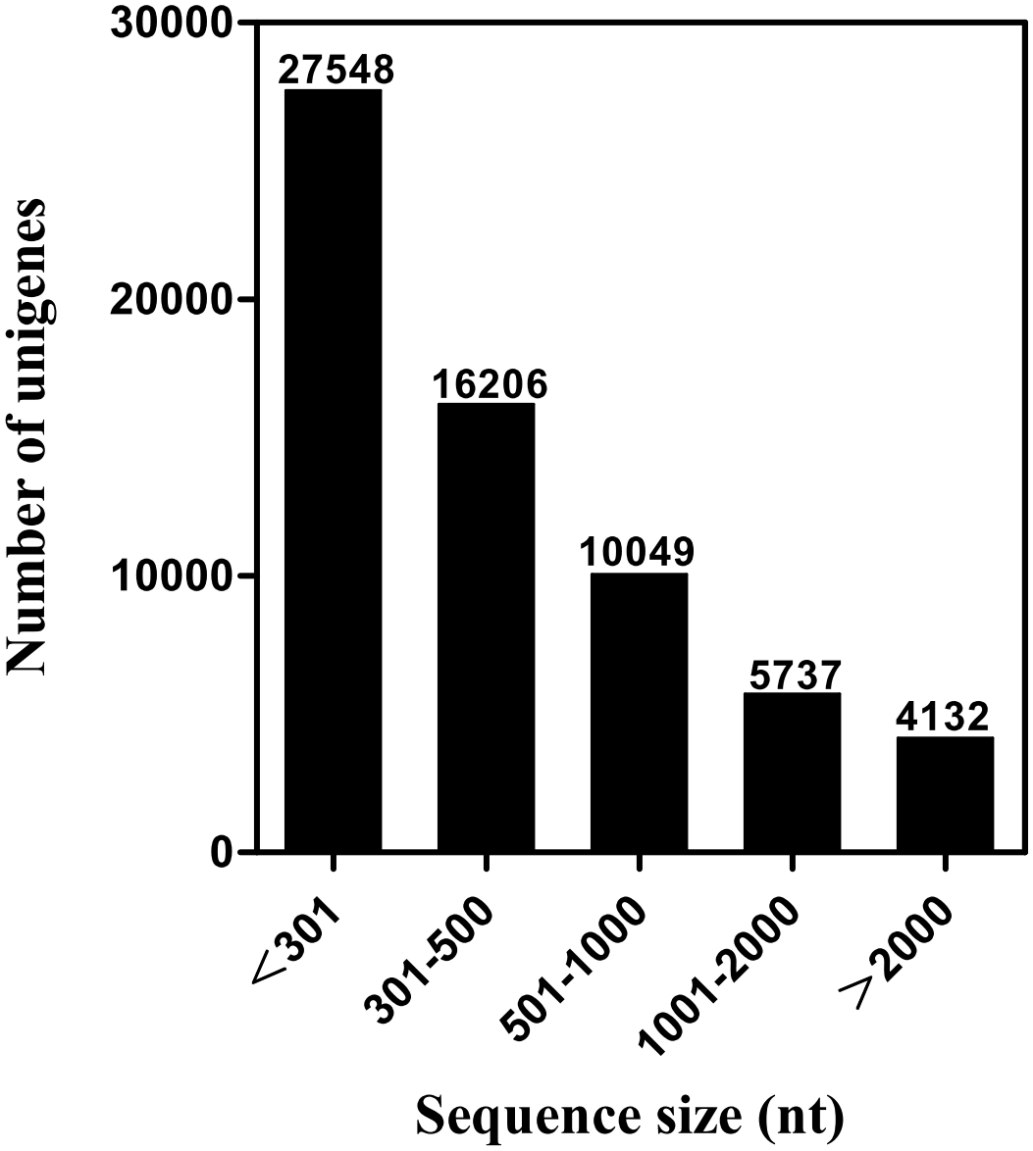
Distribution of unigene size in the *S*. *pyrastri* transcriptome assembly.

The transcriptome sequencing of *S*. *pyrastri* antennae provided about 51 million clean reads (5.1 Gb). After clustering and redundancy filtering, we acquired 63,672 unigenes with a N50 length of 1,130 bp (Table 1); unigenes > 500 bp accounted for 31.28% of the transcriptome assembly (Fig 1). As several recent publications have described [52, 53], these unigenes do not necessarily represent distinct genes.

**Table 1:** Summary of *S*. *pyrastri* transcriptome assembly.

Statistics Project	Number
Total clean reads	51,213,608
GC percentage	38.75%
Q20 percentage	96.41%
Total unigene nucleotides	41,935,322
Total unigene	63,672
N50 of unigenes (nt)	1,130
Min length of unigenes (nt)	201
Median length of unigenes (nt)	659
Max length of unigenes (nt)	27,811
Unigenes with homolog in NR	29,587

### Homology analysis and GO annotation

**Fig 2:**
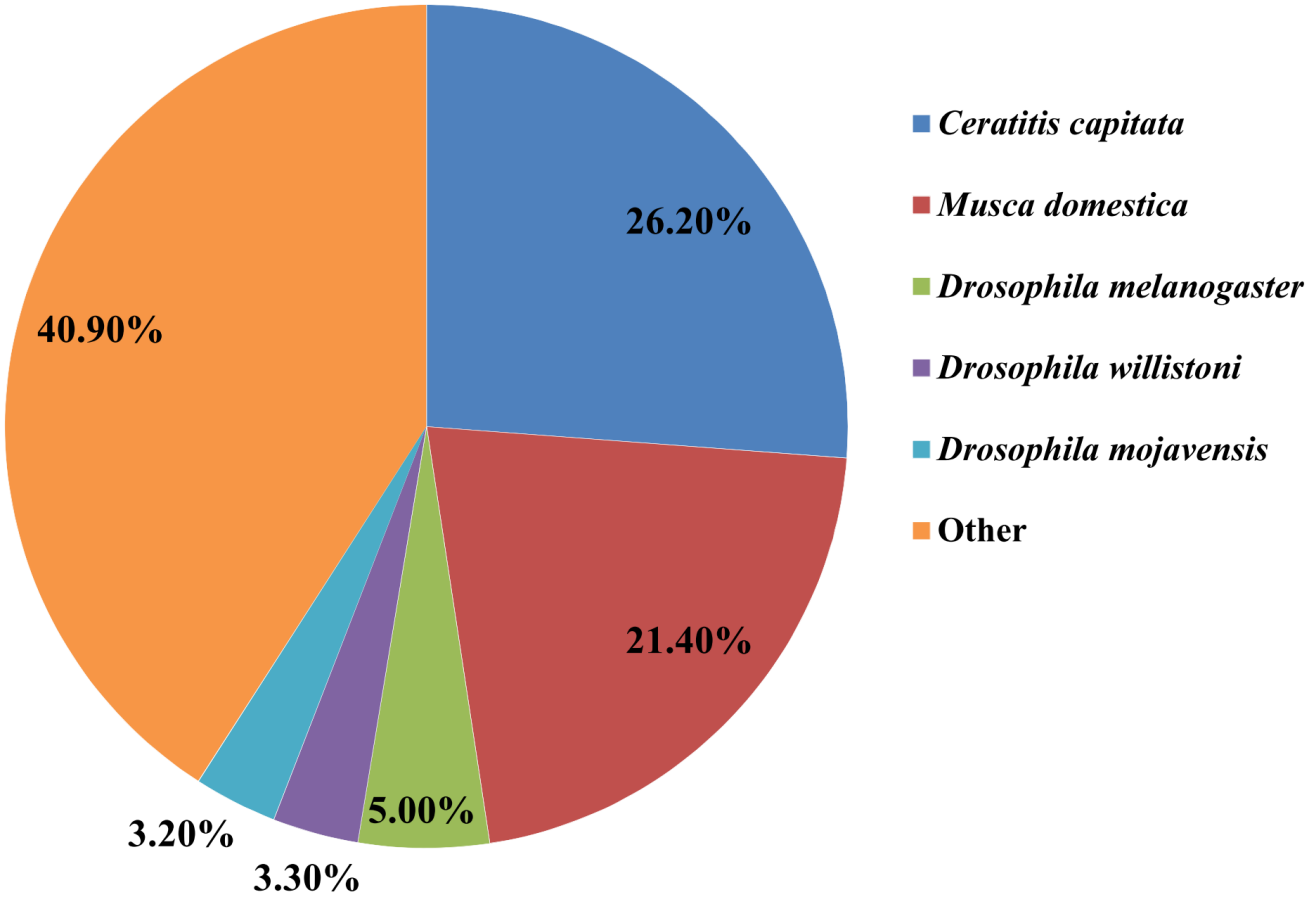
Percentage of homologous hits of the *S*. *pyrastri* transcripts to other insect species. The *S*. *pyrastri* transcripts were searched by BLASTX against the non-redundancy protein database with a cutoff E-value 10^−5^. Species which have more than 1% matching hits to the *S*. *pyrastri* transcripts are shown.

The BLASTX homology search performed for the 63,672 unigenes showed homology for 29,587 (46.46%) of them in the NCBI Nr protein database. The best match was to *Ceratitis capitata* sequences (26.20%), followed by *M*. *domestica* (21.40%), *D*. *melanogaster* (5.00%), *D*. *willistoni* (3.30%), and *D*. *mojavensis* (3.20%) sequences (Fig 2).

**Fig 3:**
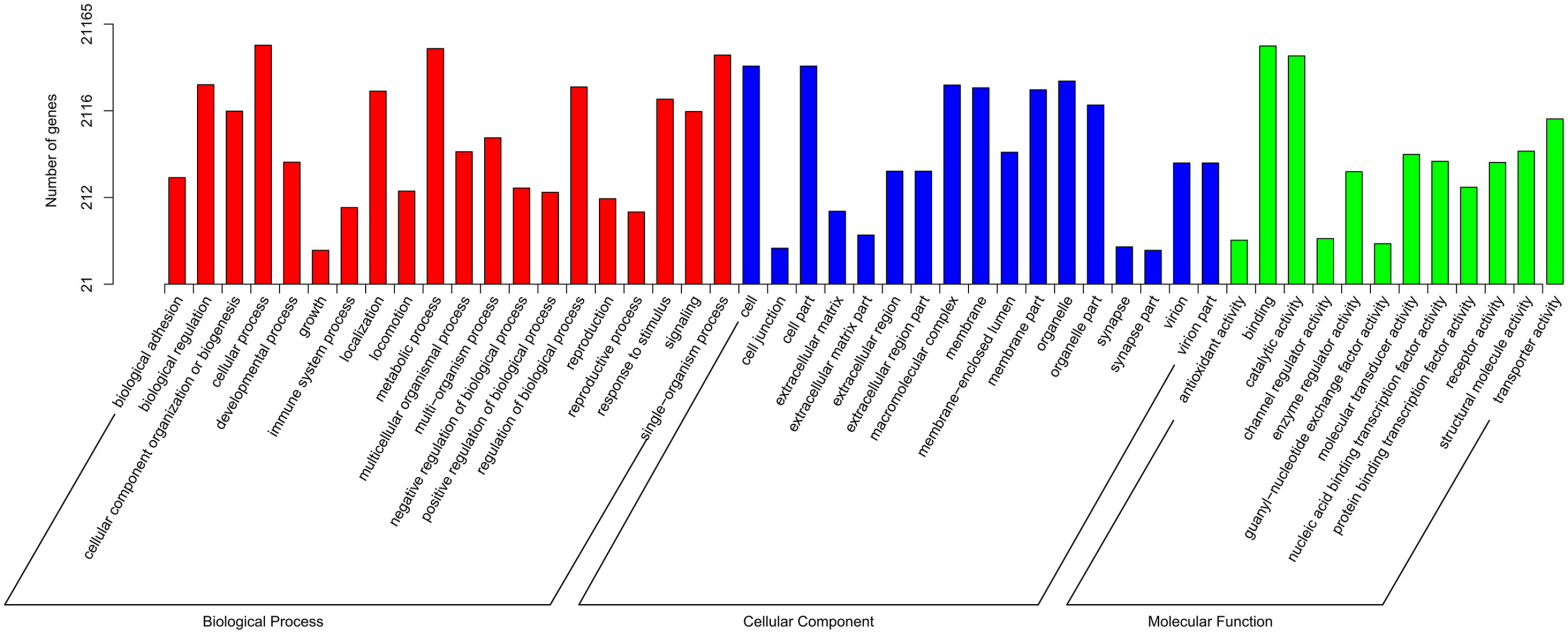
Gene ontology (GO) classification of the *S*. *pyrastri* transcripts with Blast2GO program. The Y-axis shows the number of annotated GO terms in three categories: biological process, cellular component, and molecular function. The X-axis shows three areas of annotation, and in each area the sequences are further divided into subgroups.

The GO annotations resulting from the Blast2GO pipeline revealed that 33.24% (21,165) of all unigenes were successfully assigned to functional groups. The most well represented groups were: cellular, metabolic, and single-organism processes in the “biological process” category; cell, cell part, and organelle in the “cellular component” category; binding, catalytic activity, and transporter activity in the “molecular function” category (Fig 3).

### Identification and phylogenetic trees of candidate OR, GR, and IR genes

According to the homology analysis, 60 transcripts belonging to chemosensory receptor families were newly identified in this study, including 38 *ORs*, 6 *GRs*, and 16 *IRs* (Table 2).

**Table 2:** The Blastx match of *S*. *pyrastri* candidate OR, GR and IR genes.

Gene	Acc.	TMD	ORF	Complete	Best Blastx Match				
Name	No.		(aa)	ORF	Name	Acc. No.	Species	E value	Identity (%)
***Odorant Receptor (OR)***									
OR1	KU291817	6	369	N	odorant receptor 22c	XP_011210110.1	*Bactrocera dorsalis*	2.00E-47	30
Orco	KU291818	7	476	Y	odorant receptor coreceptor	XP_005175278.1	*Musca domestica*	0.00E+00	90
OR3	KU291819	5	405	Y	odorant receptor 22c	XP_011210110.1	*Bactrocera dorsalis*	7.00E-58	30
OR4	KU291820	5	405	Y	odorant receptor 22c	XP_011210110.1	*Bactrocera dorsalis*	1.00E-42	28
OR5	KU291821	6	388	Y	putative odorant receptor 69a	XP_005180133.1	*Musca domestica*	7.00E-132	51
OR6	KU291822	5	398	Y	odorant receptor 22c	XP_011210110.1	*Bactrocera dorsalis*	1.00E-117	47
OR7	KU291823	5	403	Y	odorant receptor 74a-like	XP_005185292.1	*Musca domestica*	1.00E-59	32
OR8	KU291824	7	388	Y	odorant receptor	AID61215.1	*Calliphora stygia*	2.00E-124	52
OR9	KU291825	6	381	Y	putative odorant receptor 92a	XP_011208819.1	*Bactrocera dorsalis*	7.00E-64	36
OR10	KU291826	8	414	Y	odorant receptor 63a	XP_005178182.1	*Musca domestica*	5.00E-120	44
OR11	KU291827	6	384	Y	odorant receptor	AID61224.1	*Calliphora stygia*	7.00E-123	51
OR12	KU291828	6	393	Y	odorant receptor 67c-like	XP_004521076.1	*Ceratitis capitata*	6.00E-107	44
OR13	KU291829	6	388	Y	odorant receptor	AID61212.1	*Calliphora stygia*	4.00E-92	39
OR14	KU291830	2	244	N	odorant receptor 49a-like	XP_011212431.1	*Bactrocera dorsalis*	3.00E-59	34
OR15	KU291831	4	371	Y	odorant receptor	AID61215.1	*Calliphora stygia*	3.00E-174	69
OR16	KU291832	4	391	Y	odorant receptor	AID61232.1	*Calliphora stygia*	2.00E-153	60
OR17	KU291833	5	424	Y	odorant receptor 13a	XP_011295797.1	*Musca domestica*	2.00E-157	53
OR18	KU291834	5	341	N	odorant receptor	AID61211.1	*Calliphora stygia*	1.00E-94	46
OR19	KU291835	6	398	Y	odorant receptor 94a-like	XP_011179733.1	*Bactrocera cucurbitae*	2.00E-105	43
OR20	KU291836	5	376	Y	odorant receptor 7a-like	XP_011208898.1	*Bactrocera dorsalis*	4.00E-74	35
OR21	KU291837	6	392	Y	odorant receptor 83a-like	XP_011184142.1	*Bactrocera cucurbitae*	4.00E-21	26
OR22	KU291838	6	413	Y	odorant receptor 13a-like	XP_011185366.1	*Bactrocera cucurbitae*	5.00E-62	33
OR23	KU291839	6	383	Y	odorant receptor 67d-like	XP_011203703.1	*Bactrocera dorsalis*	5.00E-108	43
OR24	KU291840	5	402	Y	odorant receptor 24a-like	XP_011300122.1	*Fopius arisanus*	7.00E-78	40
OR25	KU291841	7	372	Y	odorant receptor	AID61210.1	*Calliphora stygia*	8.00E-150	56
OR26	KU291842	7	423	Y	odorant receptor 13a-like	XP_011185366.1	*Bactrocera cucurbitae*	4.00E-82	37
OR27	KU291843	5	401	Y	Or22c	XP_001356952.1	*Drosophila pseudoobscura pseudoobscura*	1.00E-39	29
OR28	KU291844	6	367	Y	odorant receptor 73	EFA05710.1	*Tribolium castaneum*	4.00E-11	22
OR29	KU291845	6	374	Y	odorant receptor 82a-like isoform X1	XP_011342410.1	*Cerapachys biroi*	2.00E-12	24
OR30	KU291846	6	397	Y	odorant receptor 67c-like	XP_011200401.1	*Bactrocera dorsalis*	7.00E-89	40
OR31	KU291847	6	391	Y	odorant receptor	AID61221.1	*Calliphora stygia*	4.00E-105	45
OR32	KU291848	3	144	N	odorant receptor 2a-like	XP_012157159.1	*Ceratitis capitata*	2.00E-33	37
OR33	KU291849	6	400	Y	odorant receptor	AID61213.1	*Calliphora stygia*	5.00E-108	45
OR34	KU291850	3	300	N	odorant receptor 22c	XP_011193492.1	*Bactrocera cucurbitae*	7.00E-26	26
OR35	KU291851	6	383	Y	odorant receptor 67d-like	XP_004533437.2	*Ceratitis capitata*	4.00E-103	44
OR36	KU291852	2	287	N	odorant receptor 13a-like	XP_011185366.1	*Bactrocera cucurbitae*	4.00E-48	37
OR37	KU291853	0	131	N	odorant receptor 94a	XP_011211752.1	*Bactrocera dorsalis*	1.00E-61	47
OR38	KU291854	4	236	N	odorant receptor 67d	AKI29045.1	*Bactrocera dorsalis*	2.00E-52	39
***Gustatory receptor (GR)***									
GR1	KU291871	4	270	N	gustatory receptor 28b, isoform C	NP_995642.1	*Drosophila melanogaster*	7.00E-134	81
GR2	KU291872	6	444	Y	gustatory receptor 1	AFH96948.1	*Musca domestica*	0.00E+00	83
GR3	KU291873	7	455	Y	gustatory receptor	AID61256.1	*Calliphora stygi*	0.00E+00	73
GR4	KU291874	6	372	Y	putative gustatory receptor 39b	XP_012160377.1	*Ceratitis capitata*	4.00E-40	28
GR5	KU291875	6	352	N	putative gustatory receptor 39b	XP_005191420.1	*Musca domestica*	1.00E-26	26
GR6	KU291876	7	444	Y	gustatory receptor candidate 6	AID61262.1	*Calliphora stygi*	0.00E+00	72
***Ionotropic Receptor (IR)***									
IR1(67c)	KU291855	1	123	N	ionotropic receptor 67c	NP_729609.1	*Drosophila melanogaster*	1.00E-09	31
IR2(31a)	KU291856	3	593	N	ionotropic receptor 31a, isoform C	NP_001260346.1	*Drosophila melanogaster*	0.00E+00	50
IR3(75q2)	KU291857	4	645	Y	putative ionotropic receptor IR75q2	AFC91752.1	*Cydia pomonella*	1.00E-137	42
IR4(40a)	KU291858	1	337	N	ionotropic receptor 40a	AKI28985.1	*Bactrocera dorsalis*	1.00E-120	66
IR5(56d)	KU291859	3	576	N	ionotropic receptor 56d	NP_611432.1	*Drosophila melanogaster*	1.00E-29	23
IR6(92a)	KU291860	2	645	Y	ionotropic receptor 92a	AKI28990.1	*Bactrocera dorsalis*	0.00E+00	43
IR7(75d)	KU291861	4	674	Y	ionotropic receptor 75d	AKI28987.1	*Bactrocera dorsalis*	0.00E+00	66
IR8(64a)	KU291862	3	1148	Y	ionotropic receptor64a	AID61277.1	*Calliphora stygi*	2.00E-116	61
IR9(41a)	KU291863	3	643	N	ionotropic receptor41a	AID61276.1	*Calliphora stygi*	9.00E-150	38
IR10(8a)	KU291864	4	885	Y	ionotropic receptor8a	AID61272.1	*Calliphora stygi*	0.00E+00	59
IR11(92a.2)	KU291865	4	552	N	ionotropic receptor	AID61282.1	*Calliphora stygi*	9.00E-159	50
IR12(76b)	KU291866	3	617	N	ionotropic receptor 76b	NP_649176.1	*Drosophila melanogaster*	0.00E+00	60
IR13(25a)	KU291867	3	939	Y	ionotropic receptor25a	AID61273.1	*Calliphora stygi*	0.00E+00	85
IR14(21a)	KU291868	4	577	N	ionotropic receptor 21a	NP_001097043.1	*Drosophila melanogaster*	0.00E+00	66
IR15(94e)	KU291869	2	247	N	ionotropic receptor 94e	NP_001097885.2	*Drosophila melanogaster*	5.00E-56	43
IR16(84a)	KU291870	4	645	Y	ionotropic receptor 84a	ADU79034.1	*Drosophila melanogaster*	8.00E-120	37

TMD: transmembrane domain

Thirty-eight different transcripts encoding candidate ORs were identified based on the *S*. *pyrastri* antennal transcriptome data, and 30 of these sequences contained a full-length ORF that encoded 361–476 amino acids. One OR sequence sharing high identity with the conserved Orco proteins of other insect species was obtained and thus named SpyrOrco. This amino-acid sequence had 90% identity with the co-receptor of *M*. *domestica* (XP_005175278.1), and the remaining SpyrORs had 22–69% identity with other insect ORs. Based on these comparisons, and on our previous predictions [23, 24, 54–57], full-length SpyrORs had 4–8 transmembrane domains (TMDs) (Table 2). Additionally, we identified 6 GR candidates in *S*. *pyrastri*, similar to the number of candidates reported in the recent antennal transcriptomic study of *B*. *dorsalis* [24]. Among the 6 candidates, only 4 were likely to represent full-length ORFs (SpyrGR2, 3, 4, and 6), and each contained 6–7 TMDs.

**Fig 4:**
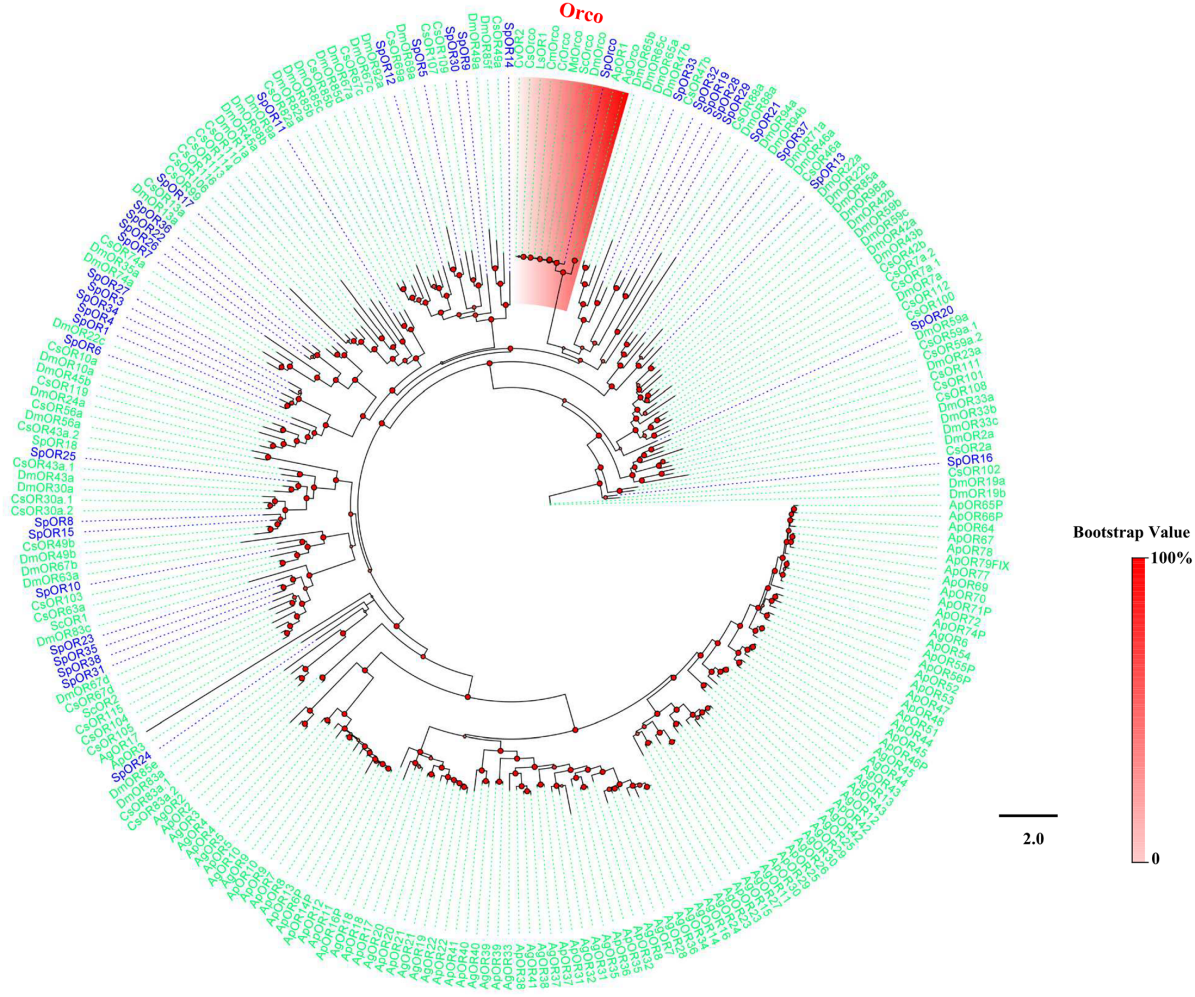
Phylogenetic tree of insect OR. The *S*. *pyrastri* translated genes are shown in blue. This tree was constructed using PhyML based on alignment results of ClustalX. Orco clade is marked in red. Sp: *S*. *pyrastri*, Ap: *Acyrthosiphon pisum*, Ag, *A*. *gossypii*, Dm: *D*. *melanogaster*, Sc: *Stomoxys calcitrans*, Md: *Musca domestica*, Cr: *Chrysomya rufifacies*, Cm: *Chrysomya megacephala*, Cs: *C*. *stygia*, Ls: *Lucilia sericata*, Cv: *Calliphora vicina*.

**Fig 5:**
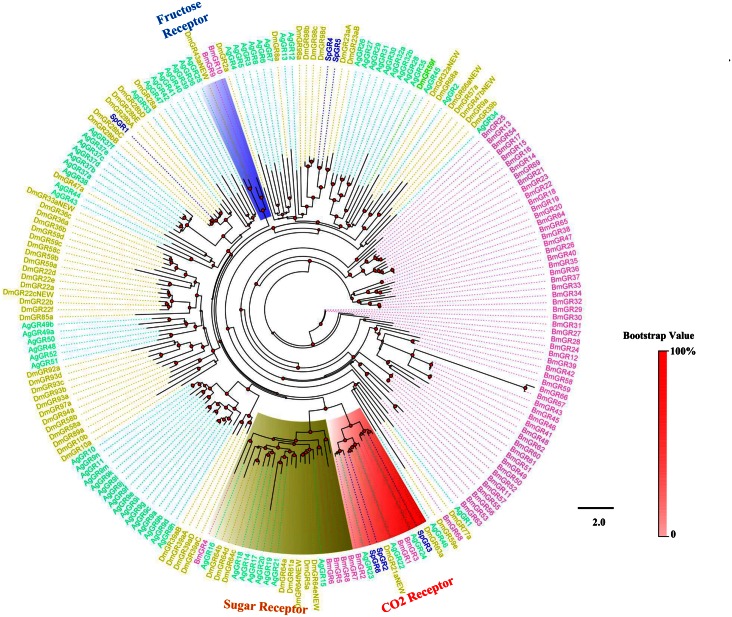
Phylogenetic tree of insect GR. The *S*. *pyrastri* translated genes are shown in blue. This tree was constructed using PhyML based on alignment results of ClustalX. Sp: *S*. *pyrastri*, Dm: *D*. *melanogaster*, Ag: *A*. *gambiae*, Bm: *B*. *mori*.

**Fig 6:**
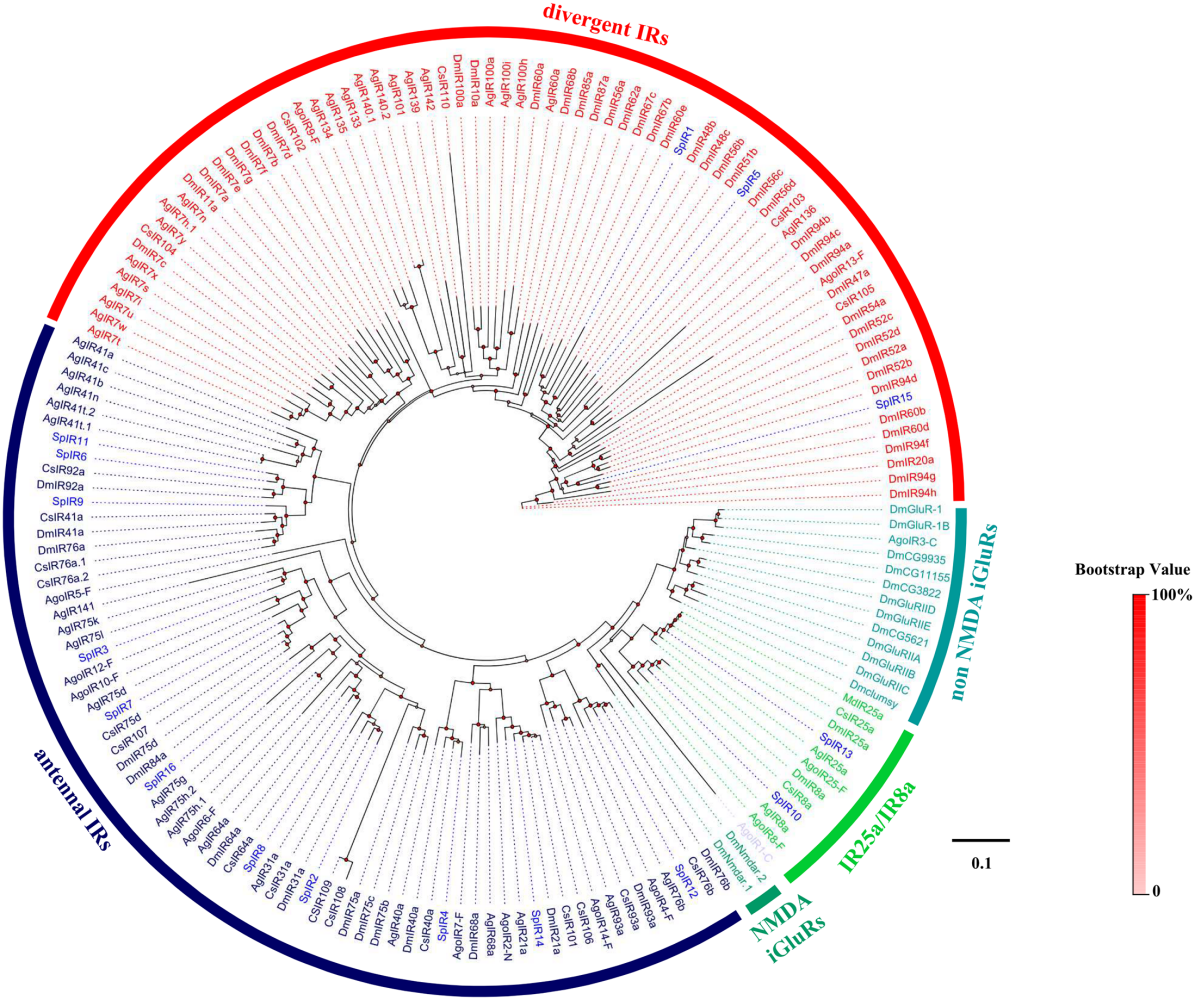
Phylogenetic tree of insect IR. The *S*. *pyrastri* translated genes are shown in black. This tree was constructed using PhyML based on alignment results of ClustalX. Sp: *S*. *pyrastri*, Dm: *D*. *melanogaster*, Cs: *C*. *stygia*, Md: *M*. *domestica*, Ag: *A*. *gambiae*, Ago: *Aphis gossypii*.

The phylogenetic tree constructed using all SpyrOR sequences and the Orco sequences from 8 Diptera and 2 Hemiptera revealed the clustering of SpyrOrco with other Diptera Orco sequences (Fig 4). All identified SpyrORs had at least 1 dipteran orthologue. The tree constructed for GR sequences evidenced that 3 SpyrGRs (SpyrGR2, 3, and 6) were distributed in CO_2_ receptors, but no orthologues of sugar or fructose receptors were found (Fig 5). Although we identified 16 IR candidates in *S*. *pyrastri*, a similar number to those found for *D*. *melanogaster* [58], only 7 of these IR candidates likely represented full-length ORFs [SpyrIR3(75q2), 6(92a), 7(75d), 8(64a), 10(8a), 13(25a), and 16(84a)], encoding 645–1148 amino acids and containing 2–4 TMDs (Table 2). Phylogenetic analysis of the IRs revealed that these 16 candidates were clustered into antennal IRs, divergent IRs, and IR25a/IR8a clades (Fig 6).

### Sex distribution and tissue expression of candidate OR, GR, and IR genes

**Fig 7:**
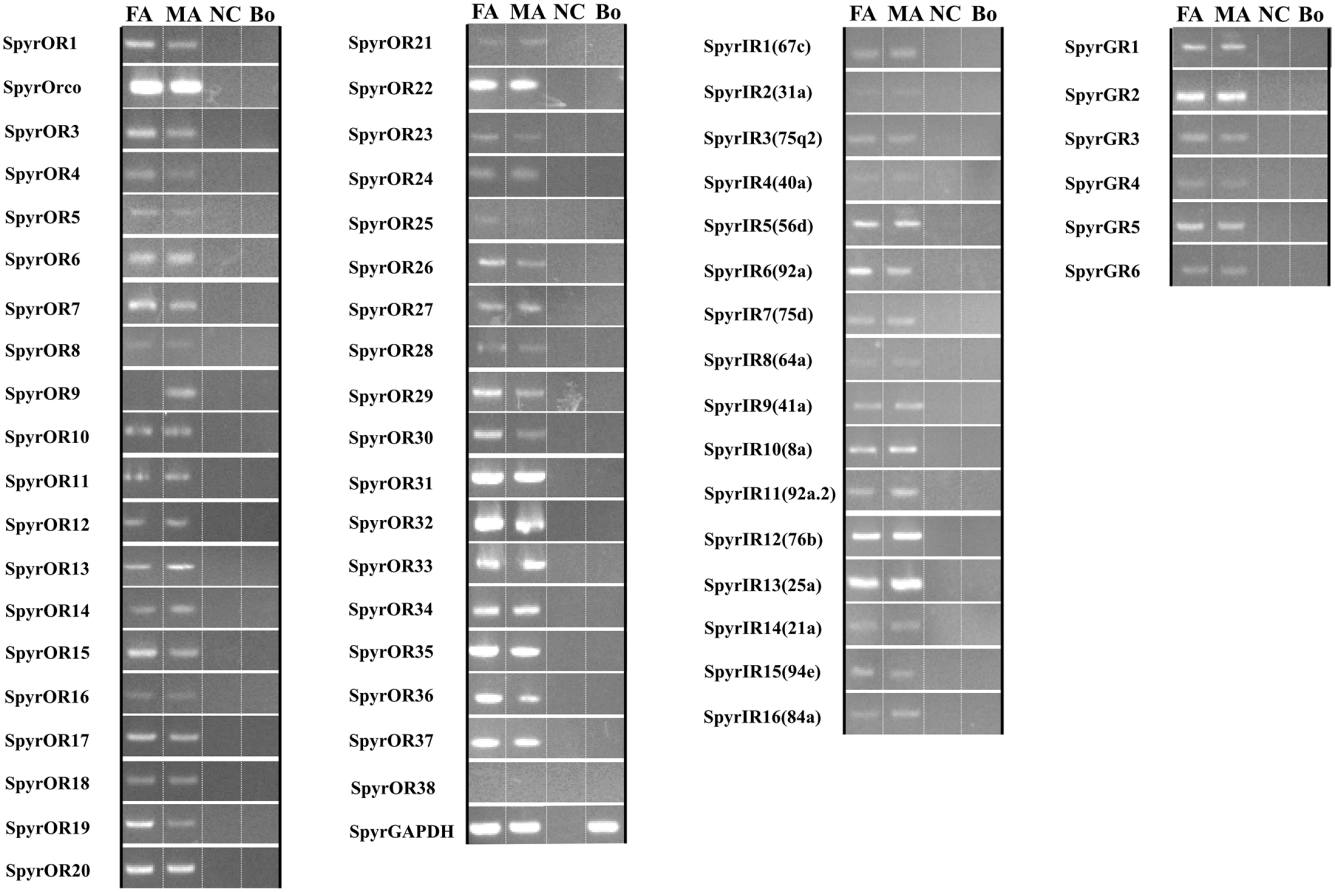
Expression patterns of candidate OR, GR and IR genes, using RT-PCR. GAPDH gene was used as a positive control and NC (no cDNA template) as a negative control. FA, female antennae; MA, male antennae; Bo, whole insect body (without antennae).

Based on recent studies, including our own, RT-PCR is a reliable method for analyzing the tissue expression of chemosensory genes in many insects [28, 37, 59–62]. Therefore, we also used this method to investigate the chemosensory receptor genes expressed in *S*. *pyrastri* antennae and body, using *SpyrGAPDH* as the reference gene. RT-PCR showed that 37 candidate *ORs* (except for *SpyrOR38*) were expressed in the antennae. Of the candidate *ORs*, 1 (*SpyrOR13*) exhibited male-biased expression, while 11 (*SpyrOR1*, *3*, *4*, *7*, *15*, *19*, *23*, *26*, 29, *30*, and *36*) exhibited female-biased expression. Remarkably, *SpyrOR9* was the only gene exhibiting male-specific expression. The remaining *ORs* were expressed fairly equally in both male and female antennae (Fig 7). Candidate GR and IR genes did not exhibit significant sex biased expression, except for *SpyrIR16(84a)* that showed male-biased expression in the antennae (Fig 7). No candidate receptor genes were expressed in *S*. *pyrastri* body tissues (Fig 7).

## Discussion

In comparison with our understanding of insect pests, the molecular basis of chemoreception in natural enemies is poorly understood. Here, we sequenced and analyzed the transcriptome of *S*. *pyrastri* using samples from antennae. Among the 63,672 unigenes, only 46.46% had homologous matches to the NCBI Nr protein database and 33.24% were annotated to 1 or more GO terms. Although these percentages are similar to those found in other species [63–65], they indicate that most *S*. *pyrastri* genes are either non-coding or homologous with genes that have not been annotated to GO terms. Importantly, we identified 60 novel chemosensory receptor genes in *S*. *pyrastri*. Our results not only provide an important basis for further elucidation of the molecular mechanisms underlying chemoreception, but also provide insights into insect physiology and natural enemy-based control strategies [66–68].

Previous studies found that chemical cues (e.g., sex pheromones, aphid secretions, and plant volatiles) play a major role in mediating many *S*. *pyrastri* behaviors, including mating, parasitism, and oviposition [33–35], suggesting that consistently expressed ORs, GRs, and IRs are likely involved in these behaviors. However, *S*. *pyrastri* chemosensory genes have not been identified before this study. The number of chemosensory receptor transcripts identified in *S*. *pyrastri* in the present study (60) was greater than that reported for *B*. *dorsalis* (40) [24], but 2.5 times lower than that found in *D*. *melanogaster* (153) and 2.9 times lower than that of *A*. *gambiae* (177) [42, 58, 69] whose genomes have been sequenced. These differences suggest a high probability of identifying more *S*. *pyrastri* chemosensory receptor genes once its genome is fully sequenced.

Furthermore, 59 of the 60 chemosensory receptor genes were expressed in adult antennae, which mirror the numbers found in *D*. *melanogaster* [70], indicating their importance in *S*. *pyrastri* olfaction. We found fewer ORs in the antennal transcriptome of *S*. *pyrastri* (38) than in the complete genome of *D*. *melanogaster* (62). However, our *S*. *pyrastri* OR count was closer to that in the antennal transcriptome of *C*. *stygia* (50), suggesting that we may have missed larvae-biased or lowly expressed ORs. *SpyrOR38* expression, for instance, was not detected in RT-PCR but was identified in RNA-Seq, suggesting that the latter method may be more sensitive than RT-PCR for detecting low expression levels. Despite these limitations, the patterns evidenced in the present and previous studies are consistent with the evolution of species-specific plant-host adaptation and odorant perception in Diptera. Remarkably, and similar to that observed in other insects [23, 26, 64, 71], a species-specific expansion of ORs was found in *S*. *pyrastri*, as evidenced by the number of SpyrORs with no orthologues distributed in the several clusters of the phylogenetic tree (SpyrOR31/38/35/23, SpyrOR7/36/22/26, SpyrOR32/19/28/29, and SpyrOR27/3/34/1/4, Fig 4). In addition, 7 of these ORs (Spyr36/26, Spyr19/29, and Spyr3/1/4) exhibited female-biased expression, suggesting they might be related to specific odor-oriented female behaviors, such as selecting conspecific males and the oviposition substrate. Thus, these receptor-mediated behaviors might be species-specific behaviors, as the cues triggering them are only perceived by hover fly females cues, meaning these 7 female-biased ORs might be species-specific receptors.

One gene (*SpyrOrco*) displayed high identity with Orco genes known for other insects, suggesting that Orco also acts in *S*. *pyrastri*. This protein is more highly conserved than other ORs [1, 42, 72, 73] and might act as a chaperone and dimerization partner for other insect ORs, forming a ligand-gated ion channel to specific ligands [56, 73–75] and having a similar function in different insects [76]. Notably, only 2 ORs were male-biased (*SpyrOR13*) and male-specific (*SpyrOR9*). These 2 genes might function as insect pheromone receptors (PRs), a well-studied group [77–80], similar to DmelOR67d in *D*. *melanogaster* and BmorOR1 in *B*. *mori*, which are essential for detecting the male-specific pheromone 11-cis-vaccenyl acetate (VA) [81, 82] and to respond to the sex pheromone component bombykol [75], respectively. Additionally, we identified 11 genes displaying female-biased expression (*SpyrOR1*, *3*, *4*, *7*, *15*, *19*, *23*, *26*, 29, *30*, and *36*). These *SpyrORs* may be responsible for detecting oviposition-related cues or male-produced courtship pheromones. Still, the putative functions of male- and female-biased genes require verification with *in vitro* and *in vivo* studies.

To distinguish candidate IRs from ionotropic glutamate receptors (iGluRs), SpyrIRs were aligned with iGluRs from *D*. *melanogaster* and IR orthologues from several other insect species before BLASTX and phylogenetic analyses. Overall, we identified 16 IRs in the antennal transcriptome of *S*. *pyrastri* and verified these were distinct from iGluRs, suggesting *S*. *pyrastri* has fewer antennal-expressed IR genes than *D*. *melanogaster* (18) [58] and *A*. *gambiae* (22) [58]. Although we might have missed some transcripts in our antennal transcriptome, the available *SpyrIR* genes provided some insight into their function. Specifically, sequence alignments and phylogenetic analyses revealed that SpyrIR10(8a) and SpyrIR13(25a) belong to the co-expression IR group. Receptors in this group are similar to Orco as their co-expression with other IRs implies they play a role as co-receptors [83]. Thus, SpyrIR10(8a) and SpyrIR13(25a), as well as other SpyrIRs, may serve the same function in chemical communication as their orthologues in *D*. *melanogaster* [17, 84, 85].

Although many GRs have been identified in a variety of insect species [23, 24, 55, 65, 86, 87], we only identified 6 *GRs* in *S*. *pyrastri*. However, this low number was expected because GRs are primarily expressed in gustatory organs such as the proboscis and not in the antennae [13]. Despite the low number of GRs, phylogenetic analysis revealed that 3 GRs (SpyrGR2, 3, and 6) were clustered into the “CO_2_ Receptors” group with DmelGR21a and DmelGR63a from *D*. *melanogaster*, indicating they might be involved in CO_2_ detection [88, 89]. Like *SpyrIRs*, *SpyrGRs* were equally expressed in male and female antennae. Therefore, the function of GRs in olfactory progresses appears not to differ between sexes.

## Conclusions

Based on RNA-seq and RT-PCR data, sequence analysis of the antennal transcriptome data allowed successfully identifying an extensive set of candidate OR, GR, and IR genes that might be related to the odorant perception of *S*. *pyrastri*. As the first step towards understanding their function, we performed a comprehensive and comparative analysis of ORs, GRs, and IRs phylogeny and expression patterns according to sex. These analyses evidenced the species-specific expansion and the sex-specific or -biased expression of some genes, respectively. Therefore, this study contributes to an increased understanding of the molecular mechanisms underlying chemosensory-guided behaviors in *S*. *pyrastri*, providing data for further functional analyses of chemosensory receptors.

## Supporting Information

S1 Table Amino acid sequences of *S. pyrastri* used in phylogenetic analyses. (PDF)

S2 Table Primers used for RT-PCR. (XLS)
