# Supplementary material for: Molecular Characterization and Sex Distribution of Chemosensory Receptor Gene Family Based on Transcriptome Analysis of Scaeva pyrastri
Source: PLoS One. 2016 May 12;11(5):e0155323. doi: 10.1371/journal.pone.0155323 (PMC4865182; doi:10.1371/journal.pone.0155323)
Supplement: S1 Table — (PDF) [file pone.0155323.s001.pdf]

OR

>SpOR1
MNRLTKIKIIFNLTLLLTAIVGEIRFAWFLRRDPLNALNVVCPYLTKMVTWMKLICFLIYRKDLGHILKCLFEHCQKD
ANTSTKRNMISKVSYFASVWCIVVYVNAFSTSILFAAKPLIIWSYQFLVNGNKDSWVSLPFQVALPYQDMTLINN
KTLYSIIYCFLAHSGTITIFGISGTDGTFFCFCMYISVSYQCLQEDFKTAFKKHTSKDLDSSSQEIFYKELTQLIRRQQN
IIEIFKSFNRIYKFMIFIHFASASIILAIVLANLLLISGISKLIYLTYAFGAGIQLFTYCYGAEYVTKNCVKLSEVLYFCDWY
KCNRRVKLLILFTLIKSQRGSPLRAPFFQPSLQLFALIIQRSGSYLTILQAVL
>SpOrco
MQQQTKYIGLVADLMPNIKLMKYSGLFMHNYTSGSSFFKKVYSCFHFTLILLQFFSILANMALNADEVNELSGN
TITALFFTHCITKFIFFAVNQKQFYRTLNIWNQVNSHPLFAESDARYHSIALAKMRKLFVLVMLTTVLSVVAWTTIT
FFGESVKFARDMETNETITVEIPRLPIKAFYPWDASSGMFYMISFVYQVYFLLFSTTQSNLCDVLFCSWLIFACEQ
LQHLKGIMKPLMELSATLDTYRPNSAALFRSLSANSKSELIINEEKEPVDTLDMSGVYSTKADWGAQFRAPSTLQ
TFNGTNGMSNPNGLTKKQEMMVRSAIKYWVERHKHVVRLVAAIGDTYGAALLLHMLTSTIKLTLLAYQATKIN
GVNVYAFTVIGYLSYSLAQVFHFCIFGNRLIEESSSVMEAAYSCHWYDGSEEAKTFVQIVCQQCQKAMTISGAKF
FTVSLDLFASVLGAVVTYFMVLVQLK
>SpOR3
MSFIKFSRFDPLESAFFRMAKWTLVFLGIWPLDLLNRLTIFKIIFNSSALLTAIIGEIRFAWFIRSEPLEALDAACPSLT
KMVTMIKLSCFLIYRNQLATLLNELYEHLYADTKNEKKNLLIGKISFFTSVWCLMLFLLSLSTSFLYTVQPIIVWIYQR
FTGTKGNWVTLPFQAALPYQDMTKLNNRFVYSLVYCFLAHSGNVTVFGISGADGIFMCFCMYISVSYECLQND
FKTAFNTYCSKNSEPESNGDDELLYNELSFLVKRQQKIIEMFEHFNQTYKLIIFGHFLFASIELGIVLVNIVLSTGITK
MIFLTYVLGSCCQLYTYCYGAENVNQNCVKLSETLYFCEWYQCNTKIKRLVLMVLMKSQKGSLLDAPFFTPSLPL
FASIIQTSGSYFTILLAFM
>SpOR4
MAPKLLQFICSDSFEVSFCRVTRWILRFLGFWPLDSLNRLIIINIIFNLILLITACIGELRFAWFIREEPLKALDAFCPC
LTKTVTWMKLVTFFVYRKDLSIILKSLFEHCKTDTNNLTKRTMISKTSFNTSIWCLLLYINAFIVSFLFSLKPLLVWSY
HFYVTGHRGNWVALPFQAALPYQDMTQINNKAWYSIIYCILAHSGTITVFGITGCDGTLFCFCMYISISYQCLQE
DFKTVFNKHMSRDLKAVRNDVLYNELTELVKRQQNIMEIFKSFNRIFKFMIFIHFGSASIILAIVLVNFLLIPGISKLI
YLNYAFGTAIQLFTFCYGAERVNQNCLQLSEALYFCDWYKSNRRVKLLILFVLRNSQKGSPLEAPFFSPSLILFSSIIQ
KSVSYLTILQTFL
>SpOR5
MEFPNLAFKLAFIKPFRRPHETKPTILAWILFSIGAGNLVYQNFGMLIAMIFTKNDSIDIVLIISETGSILGLAMVAL
CKMTILFAYRKDILSILEELENYFPGVLKVHRCCRRIQYRVKYFAEFSRKMMKITTIFFMFAFLVYNLIPVGQSLIEW
LVFGLHFQYRYQSNTWYPWNGNTVIRYTLSYVCQVYSSLAGVAFIMAGEFMLCFFLTQMRMHFDFLARALESL
DAVEVEADPGCMNFLIQYHTTLIRLTTEINHIFNISFCVNFATSSVAICLMGCSMVMGISLISAVRYSIGLLSFLVFT
LFICYNGSIFTQTSAKLHSAAFYHNWYDASPKYRKTILIIIMRAAKPSELQAYKFSKVSMETFMDILKFSYKLFTFFR
AME
>SpOR6
MKFRFLSPDTPIKESYLLIPRFSLRVVGCWPQRKVPTFIGILHFLASSSAVAFGAFGECIAGALTLNQILETMEAWC
PGVTKQISLFKMWIFFVHREKLYSMIERIEQLLTADSNEDKKKIAQKLSVIASILTVILFVSANSTNLFFNTRPLINNII
RISNGKNATLDLPFKMILPDVLTHYPVYPLTYISMSYSGVMTVFTFCGVDGCFVGLCFYISALFRMIQYDIQELFG
HLQYHEKAGRRDNQIMRQELKVIIRRHNEVIDLCNEFKDIFTTIIMGHFLSASLVVCFSVIDLLYNTGIGLVLYVCYS
IAALSQLFLYCIGGTYVNDSSLEICDSVYLVEWYKCDAETRRMILMILIRAQRPATINVPFFTPSLPTFMGILSTTGS
YIALMKSFL

>SpOR7
MYYQPTLPDGTPITLPWQLRSFFSNVLWPLQDNPTPKMKRIDNIFVGFTFFSFLLAVEAAIVFFINNLSDIFLSTE
AFSNLVTYNDVILRLFNMAWQKNALKKLLKKFYAEIYVDEKENSEIHKKVQKIIGPINIYAWVYITSFLTFVIGPIIAA
FLYNTRLLPHKLEFHFSLENPFVYAWIMAQLTYNGLCASLFVGGEICLLGNFVGYLCARFEMMQEELEEIDDFLIS
VNDSSKLAGKFLKIVDKCIARTNKLIEFAEEIQKVFSFQFFVMTAHSTLMLCVVIFQTSILDFGSAKFISYVLWMVA
KVFELIILGGLGSRLITTTNNISSMFYHCNWEQIVFRSTNRKDNIILMKKINAAILIYHNQIKLNGFGFFSISLEQAA
SLIQGAGSYFTVLQTFS
>SpOR8
MVMVLEKKDPKELILQEILAIPMVAMSIRVLKYWGLIYERNWRRYFSLSLTTIFNLTQFIYMSLTREAVDVIIRNAY
MLVLWFNTILRGYLMVMQKSKYEKFLSDFIDLYKSLLHSNDKIITDMLKATNALGKNTAIINMVLGLMTVLGFCA
YPIFADTRMLPYGSHIPGINELEDPAFYIFYVYQVIITPIGCFMYIPFGSLVVSLIMFAILMCKVLQHRLRGINGDQN
SSNKILMMKIKWCIKYQKTIIKYVSIINNLTTYVFLVEFICFGTLLCGLLFLLNIAETTAQVTIAVTQISMIFSQILTLYW
YASQLAEESGKVADAAYDVNWFLIEKPMQTNILMLIMRAQSPCVMTVGNIYPMSLEMFQSLLNASYSYFTILR
RFYD
>SpOR9
MKNKNVEKYFHGTIYYFRTIGFDYENKNKLMHYYLMAGIINSAIIIFGQISFNIFQEVNLQQRTTNLSYLNYYLVSS
AKLIALFFYRDTVNDVLNEFRQIYPSEDLEKAYKLDKYLRQSLMIEKILAIFFEVVTIFYTWFPLVLSVLDFIDSGKFS
FRLPYMCWYGFNFAKYNYWFYGFKYLLQAHATWVSAITILALDLCLFSSLLQLCMHFDIINKNILSIEAAKDEEAT
ALLKEQVAYHQKLLSLAEKLNAVFTPSVLFSLLSSSLIICFTGFQLLGDITLFIVVKAIFLLAYELKQVAITCFLGDKLIEI
SLKISDAIYEHNWYKGTPKYRRLVLMVLMRSQHSVALHIAGISDISLQTFRQVMSISYQIFTLLRTG
>SpOR10
MPLEDFKTLKEKNKQRLLYLINVTYSLGINITAPSKFKDNLRIISVYLLVTSIFTLYGHVQAMIRNFGNISLMSESICLF
FQILISFLKLSYMAVFQRRFFVLLNKVETHELVNGFELLSMETPYNKELRKDAIKILNASWKFGKIQFYFYMWACV
MIFSYYFVVNMGTNIYNHAVGTANYTNVVPFSTLYPFWEDKLNQYPHFPILVLLTLSGASIAPSVALSFDGIFIYLA
LHGAALIQILKKAIPLTTSAKVPKVLRLDYLLQCIRQYEITYRYCTEVNGLYLHLTLAQFLLSLIILGVVMFQGTVGLE
TDYIVFIRMVLYISAAGYEVVIYCLNGQAIISESDGISNAWYECNWYNESIEFKQLIRMIIMRSNRTIIMRASWFST
MSLTTLLNIMRTSGSYFLLLRNLAS
>SpOR11
MEIGLQIFNRQTKCLRAMGHSLIHQVQESKWQRIPQFFVLFLVVSAQYPMINYAIFHKDDLELVTGCMSIAFTN
MLSSIKVLTFLINKIKFLEIINELKTMWQNSGEYGFNYIKGSNIMADRVVNFYFYSVFFTGFYFLLTPLAKIAWAKIN
HMPIIRELPMPMRFFFDAEHSPVYEYAYIYTGCVTVIVVSYVVAIDGLFIAFAIHLRSHFRLLQHYIENNSFKSNEA
AINTNIRSYVKYHSKLLRLAEQICQTFKPIIFVQFLITSLQVCVLVYQLVTNMNNVMIFVVYVAFLVSILIQLLIYCYG
GEMLKTESSMVATSVQISKYYNLSPKYRKVLCLVLMRSQKPVIVKAGFYVASLENFMTILKAAMSYITLIQSLEQI
>SpOR12
MAFIENFELIRIPVEFYKTMGQDIFETAKHNRLKSISLKILLYGGLGNYIIEYMLQVVFFVLALRSNEYLVAYAVAPSI
GFGLVAYFKMMTLHMNRSSIRDILREFELIYPKTMELQRDYQIEKFYRLLKPIMKLYAGLCLMNTFYYLFFPLVKSV
SACLLFGVKFDFPLPLFIWYPYDPKGNWTLYLLTYGMQLYGSLLTAVGYLTADLFLVSSVEQLCIHFNYVSLRFEQF
EPSSDENNHRYLARIVKHHIKCLEISDSVDRIFGFSLFLNFFASSGTLCFVGFQVTSATIEELLQNIVLLVTSLSQVFII
CFYGNKLMYTSQAVGEAAFGHAWYMCNMEYRRMVALVITRSQKMAAIHAPTFPPISLETFMKVISMAYQFFA
VIRTTSDN
>SpOR13
MNYSDHHEKVEQFYNVQESLYRKMGGFRLSPNSSKIKKIAYEFYGILAMMVIGVYNSSLFISFITLPSILDKIMKAF
YMLGTLIGAMVKYICVKRDYSLYRDIATDLHQDIFKPLNAAENDIFVRNLEMSIRVRNLYGYVSCLSLVYAFGLQQ
VLNPKELPAQLYAPFDMEDSTNFLIMKVFSFISVGYLCFINIAFDSWCTSFMLFIQGQLEILEYRLERIGFNGDSDL

EINLELKSCIRLHAAVHDIIGKLEIVIAFPNSLQIFCSMLVLCCNFYAISFTSVSEDTIAFLKFSIYQMAMLSQVFFICY
FANEVTLASNKLSHALYSSNWITWNKINRRLVLLTMLRFRDPIRVKSLNRCYHFDLAAFTSIVNTSYSYFALLKQM
NN
>SpOR14
MPGIKSMYQYMRFDEEFSYETPFWVEHPFYYRTKLLYGLVYFSELWGGHLCVSCNIGIDLLLCCLSRQMSMHFK
YIGQKIERYCPTGGPGDNVFLSKLIEQHQLILKLCLDLNEIFGISLVFNFFSSSGIICLSAFQVTVEGPGMEFMKFFLL
LVSALGQLFIVCSFGNELIESSLSIIECVFNHPWYRGSQSYKKQLVIFLLRCQKPATLIAKGFSVVSLVTFKQVSSMSY
RIFALLNTFHAE
>SpOR15
MLEEIQVVYMSVRILKFWSLIYDHNWRRYLCLSMTSILNITQFNYMFLTHESVDAKIRNAYMLVLWSNTIIRAYLL
VHQKAKYEKFLKDLEYIYYEIKKDGDKYIQSLLDETNLMGKYMSRLNLFLGLLTCIGFGSYPLFANEKVLPFGSIVP
GLDEYKTPFYEILYVYEMIVTPMGCCMYIPFTSLIVAFIMFAILMCKVLQYRLRNLKNPGDSNEVIKTKLIWCIKFQ
KTIIRYIQTVNNLTTYIFFLEFMAFGILLCALLFLLIIVDSLAEAVIVCAYITMIFAQILALYWYANELYQQTLNIAAAAY
ETNWFEFEIPTQKYLMLLILRAQKPCSLMVGNVHPMNLETFQSLLNVSYSYFTLLKRIYG
>SpOR16
MSTEIKLDPLDKPTLHTYEAFKYHWYVWQFCGLKFPKNAKWRVPYIVYAIVLNITVTLFFPLTLIVNCFLSENFTIL
CENLYITITDVICNLKFLNVFIVRSKLLEVEHILDRLDKRSSSEDELQALKNGMRSARKCFNIFATMFGCAIITSQLVV
YLSKERILMYPAWLPLDWKNSYQDFVIAHSYQVYGLTVQAIQDLGNDTFPQAYIRILCAHIEALSLRISKLGKNKG
LGSEKVNRELLIECIKDHQVIIELYTTIQQSISTACFAQFMCTGLAQCTIGVYMLYIGLDISKLLNTIIFFTAVTFEILILC
LFGEQLCQRAEQLTAAIYSCNWMDQNREFKKALLLLLKRSQRPLVIMAGDIIPLKLPTFVQVMKTAYSTFAVLSEV
K
>SpOR17
MYNPRPKDGSEFKFPLQCLWLKLNGSWPVESKNQSFKRAYNTWSWYVVFSVGITIYFQTVFLIFTFGDILKTTE
NCCTTFMGALNFIRIVHLKMNQNEFKLILRNFVKYIWIKKGSNPKIETECQSNMKIFSLVSIMMMCLILMYCIMP
LIELFSNPNLEEKPFPYKMIFFFDANHGWKYVITYIFTSFAGMCVVTTLFAEDSLIGFFVIYTCGQLRILHEDCSKIM
PNAHRQIIADYGKLPVSEVHIQRKYVQQLHKIAQRHSEIINFAHSLETFNSPHLLANFSISSILICMVGFQLVTGKM
FIGDYLKFMVYILSALSQLFILCWNGDNLIQRSLETAWHLYACNWEGGVFKLDDTGKQDPISYCPAGRDFNAKL
QLMIMRSQKPLQITAMKFSVLSLQSFTKILSSSMSYFTLLQTMLEK
>SpOR18
MIIPILFNISEFIYLIVNWGNVETLILSSFMTAILFNSIFRIVSVLLNQKDFVKFIEDIQDWFQETENQRDIASLRVLN
AVAVKADTFSKLSLFLGSVGGVMTIIVPLCMNSKELPYPVWIPTVDVYQSPCYEIIFAAQVLFVMPFIIVTYIPFTN
LFINYLMFGIGSLQILQNKLENVIDSTKDFEQIDRDLEYCIQYHNKIIRFCDTLSTLLSLTNLMEFLMFSLMLCVLLFF
ILIVDSYMLQINAAIYIGSIFYVVLLSYWHANEFSYESLKVAEAAYNSNWTICNSKIKKSILIMILRSQNALQISAGG
MYPMTLETFQSLLSASYSYFTVLNGMNN
>SpOR19
MEKFSKSFSKYVFRSDPSVGRLGSIDYNLKLLQWFGLPLFGSPGEFNRPLFICGLIFLIFVRLPYHIFEIYDLITCWRN
FNDMIQNVCMSFLHLGMTLKMIMIFGRLQDFYKIIDTFRKLAKKYVKSDKQKDVFSWMEFESKISLTIYGIIPTIA
SSYGFCLVMRNPSGVVGHNLPYRAKMPEFAPSTLQYFYIPLSNAIIGFQIVGIDYLNVAFINLIRCQLRVLNISFDQ
LNESKGPVTVDPYNHLKKIIEHHTIILDIRNQIESIFKFPMLVQFIGSLLIIALTGSQALIQKETSGLVMVYLYTGCILA
ELLVYCWFGTQISEQNETLALRAYATTWYNFDVKFRKSLVILLMNAQRPFCFTAGGYLDLSLMTFSRVISKAFSIM
AVLLQEYSDG
>SpOR20
MVMKLDTTVAFSSVWRYWSFFGIHPFAINKNLYILYSVFINIAVTLLAPLQLIVQGFFVESFAELMRNMSTSFELA
ACSLQMWVLLIYRKNLLKSHQYLKELDNRVEEDPVGRMYMEQVVRNCQRLFIVYLVLFWSTVGTYGISAALAG

RLMLEGWLPFDWRSSRTLYVVAFGYQLFDSCVQCMMNLTTDIYSVFYIYVILGHIQCLAKRISNIGHDNGAKTT
EENYCELIRCVNDHRNTLDYFCTLSPVVSATIFIQFVVAAFVLCITALCFCIFDDLSFKVTSFCYLTSILIEVLPCCYLVS
EIMEASTNLTTAVYSCNWMDQDVKFRKAVIIFMQHTQKPNSITAGNMIPISLPTFMGLIRLSFSIFTLLSSF
>SpOR21
MMPRPANGILAAQKYSFYLLFVLKFPTMSPQTPKMLNFLWECVSWISVFVPLHMSFCFFKTVYDGFVLESMDT
LSNIGIQGIWLFCSFITMLNYRLKSKDLNDIIDTIEREFLWRSAKGRTYVDMERIHWHAKIVSIVWQFLPTSTVVG
SLLRPLFLKPLELPISIWYPFDKNNPYQFALAYFFQAWAQIIVVCSFAVGSSFFFSFGLLCSGQFNITRCALKNLIFTS
GVYKRFPELLQNTYQPDSAEYHLGSEQIDKLSGIGSSENFVLEDFESTRRSLPQNFSAEEIGNFRVALKNAVRHH
HLLIRLCGMMENFWNFFVVLRVLEVTLQMCFMAFVYVRTSDIFVLLFGVLAAVDLFLLTYPAEYISEQNNLIGTS
LLMGHCLQLERFYL
>SpOR22
MKFFIPTTARGDKIESIMFWLLKFNCVWPLAKYCPEYQNFIVNCLVWILLSGFFATFYAEYLFIKVNQKDISQVAE
GLCALIIGTQGIVRVLHLIFRRNKMRDILQKFYKQIYIDKIENEKLHKNCENSLRLVYFMTWSFLLTLFFIYLSAAIKV
ARDPKSNSKPYLVKMEFFYNAQEPWKYAFTVIYTGYVGFCTACIISAEDFIVGTTLTHCAARYSILHEDLAQLYEKS
LITFTSLNQQKADEIYDAFRANLKVIIKKQQNLDKFFQELQSFLSLPIFSLVLFGVFLMCTVAFALQRNGLSLETFRY
FFWLVSICLQFLMIGSFGQRCTDAAAQMSESYYMCKWEFLLFYGDTKANIRLIKDISFGIYRSQKSLHLNGMNFI
DLSSKSIGSAMSSSVSYFMFLNEMDKIG
>SpOR23
MRPSEKFAKVLRVVRLCSAVCGCDIINEKYKINNITRAVIFCITVYFIFSAYTVNLKIAENWSVVLEAFCMVGSVLQ
GISKLTSGIIYKNYFRFASKFLRQVYSEYENKGESYLNILDKCISRTRAVLKSVAVLYAVVVISLISVPIVVWIFTGKRFF
PMQFFIPYLDTNTLLGYFATIGMQSVCLVFGGFGNFAGDMFLMVFFGQANMLVDIFMLKGQELSELAVDSKDE
KVQHKLNEVIEWHQTYSRYIENLDVMYFWINFTQITTSAVSIVLTLAIMFIGEWPGAYAYILISFTMLYLYCGMGT
LVELANDRIIDEIYNIHWYILKVPQQKCIRYMLMKAQSPKEISVGGVAQLSVSTGLQVTKTVYSVFMMLLNFFDD
K
>SpOR24
MDEIIPDMDWNEDTTYAIGGYRRFCSYFGIWPSKDRFSSGLKVTFYMVLQTLTVGIFLQRLVVYGNCGTTQEIV
DTFTVSMSSTQIGLKCIFIYMQHDRFKNILDATVEDWALTTDKESKSIMLKFARIGRNIFYCQCFLGLFTAVPLILGE
IPHQRELVSELDNSTTLIRNIILAPNCWVLQSSSTSTYYLFWSLLALYVFCVAMATVACNLIVYGFGLHVYVQFELL
YRSVQELDVDKTFKEQRRIVKKFIKRHNRMLDMANELEDASTAVIFSEVSAIVIVGCIAGTLFLLAMQSGDRAVLT
AMAGRIFLVFIQLFIFCYMGEKLSIQADKIQNAIYNNCPWYEFPPQIGRDLKLIMIRSNYPFFLTAGKFYIMNLLNF
KEVVKSMFSFFSVLRLMLQN
>SpOR25
MLFENIPILSINVKIWKFWSVILSHTWRRYIFVGPIAIMNVLQFVFLYQKWGEVDTFILNAFYAMAIFNSLLRTV
MVIFNRARFEKFMTELAALYVDIEESNNQSAIETLKKATEESRKISTFNLTASFFDLIGALLYPVFSKTKVHPFGVAIP
GIDVTLFPFYEIIYFMQLSFPVILTSMYMPFVSLFVSFAMFGTAALKNIQMQLRNLYSLNQSEEQMYHDLIACISY
HARIMRFVEDLNSLVTYILFAEFMIYSVILCALLFCLNIIESVMQKIAVVMYIMTMLYVLFTYYWQANELLTESIKV
SVAAYDVEWFHCSGRFKKTLLCFIMRTQKPLVIMLGNVYPMSLETFQSLLNASYSYFTLLREAYK
>SpOR26
MKFFIPITAKGEQLKSALFWLFKLSWVWPMDPDSSGYWTLVVNCLALLFLSCFFGTVYAEFLFITEHEGDIAKIAE
CLCTTFVGTQLIIRILHLIVRRNQMRDVLQKFYREIYLHNTEDEKRHNDCESNLRLVYFMSITFFLTLFFMYLSVAIV
VARDPTSRSKPYLYQMQFSYDSQLPWNYAFTTLYTGWVGISTVTIISAEDCLIGITLRYCEMRYTMLHEDLEKLYE
TSLKSLVTKKPTLYHQTNDLYKIFNQKLKVIIGKQQGIDRFFEDLKSFLSIPIFFVVTFGVFLLCTVAFQLQKNDLSVE
TLKYFFWLVSQCLEFLVIGFFGQRCTDAAAKMADSYYMCNWESLLIHNDSKTNAKLIRDITFAIYRSQKSVHLNG
MNFMILSLDSIGSAMSSSVSYFMFLNTMEDISNKKNANK

>SpOR27
MTSSDLSDNLVEVYFRMAKWVLGFIGFWPLDPLNRLTILKIIINLIVLLTAIIGELRFSWIIRDDLLEALDALCPCLTK
LVTWLKLVCFLIYRKELAILLNDFLKHIKEDTKDKLKNSMITKVTFLIHIWWMILYFFGISVNCLYVFKPIIIWTYHRFI
NGINDKWISLPFQAALPYADMTQLDSKFLYSIVYIFLVHSGNVTIFGFPGADGMFMSMCMYISNCYQCLQEDF
KKTFKDYSQTEINVKINNIVYGKLSAIVKRQQNVIKMFNSLNHVYKFMIFGHFLFASINMGIVLMNVQLTTGMT
KIVNITFVFGACTQLYTYCYGAEHVNTNYLQISETLYFCDWYRYDKRVKSLINFVLLESQRGSLMSVPFFPPSLPLF
SSIIQTSGSYFTLLQTFL
>SpOR28
MFQFLRIWSSYRKTNKNRFDILLNSWVIIFCGSFIGIISLIDAIQAIQMDQIDVKFTKLLFLCVVLVVIHKVCTVTYN
QKIIVEFIEQLQKLSSTFENKTNFYNAHKLYKKIFFFGIYSMNISVFVFMVAPAVLFQNKKLFFQPKISFLNLQESPT
WELMFVFQSISAFIFTNITFGYDTVYCGIMITSAAILKSIAREIREVDGVIEDHQISEEKLKNIIEKIIEISGVTKNISDI
FAMTTMVHIVYVCGTMALGLYVMSIQPLNDLTCFSMLLATIMSIFLLWIYCYFGQTLVDQRRMLQTELYFLKWY
EAQRSQKIMILVTISVLQRDCFVYAGKIIPLSMETFGQVLLKAYTFFTFMNDIKYK
>SpOR29
MENLNFFKYSLDIFKFLRLWNKRVNQEKSRLATISLYTMIVINGSIIPLICISDILRSARTVEVPVLLCKALFGLAYPVC
IYKLISVTIYKNEIYGCITQIEALSSRFQVQKDIKQSLETYKKMIFIGSIAMNFCVLLVLVVSILNNELPFRSRLSVLDLN
QSPTYELMFFYQAIALTTVANLTVAHDTLFCGLLSICAAILSSISREVRDIDVRWENGGNPEIILKEIIEKIKETFCIIER
VSNVFSITTLTNILYSSNQMALSVYLVSVWGLKDLTGMLMVIIALIQTFDLWIYCYFGQIIANQNEKLQRELYCTN
WYNHSKQFKSMLLLTIFVTQNDRFVKAGRIIALSLATFSDFLKKAYVLYTFLTEVR
>SpOR30
MEKLGRNLQLSDFTKIPFDIYRLVGIRVYKYDPDEKQPLKEKIAFSMAILNLTIHFFLKNAVPFYHKFEDPIELTAFAC
YAGFVINSLCKIIAVWCGRFFLKEVLVKMEEIFPKTEEDIIQYRLDEYYKKIEFYMRTLTIYHFSTTTMFNFFPLIQSIV
EKIITGEDFVYRFPFILVYPGNEKQFPIFLFAYFYQIMAAYILSCFFLGSDLLLVSIVHLACMHFDYLGKKITDFEPTGT
DRDLIDLGKFVKYHDDTLSLSFKINEVFKVSILMNYIASCGVICMIGFQITAGSEILDMLKFFAFLISSLVHVYFICSF
GSNLLDVSGNIADAVFSHRWYMANNKYKKSLPLLLARSHKPAFLTAFKFFIVSMESFKSLGTTSYQFFTLIKTKYD
EEE
>SpOR31
MSNCREKFNSIFMIARKCAVLVGADVFNRNYRFTLMTYFVIAATNTFFVLSGYTIYVAIYIDHDFTAIFQALCMVG
SAVQGLSKLYSFTKHRVIYRKIFDEVDEVYKIYQEKSIDYQNALSNATSIISKFLKFNACLYLIGTLVIISFPISYLIFFGEK
IFVMQFLIPGIDPKSDIGHLILFVAHSTCILMGSFGNFAGDMFFFVFLLQVPLFKNILRIKFEDLNEVTTDMEIDNS
DQTLPLLRDILSWHQKYSDFTKTLSDVYFAVLFIQTFTTSFNIGMTIFITFMGAWPAGYAYLIYSFIMLYTYCGMGT
VTEISNDDVCHYVYNTCLWYRLSVGERKMLVIMMMKAQNPTLLTVGDIMPLSVTTGLSLTKTIYSFLMMLLNFT
EEEN
>SpOR32
MPLFGAQSETKLSIIFYGIIVWTLMRIPYQVFEIFDIVAIYGNMGDLTQNLCMSLLHFGASIKTIMIFSRLGDFYDIV
KQLRIMSIEYCKSKEQRDVFFKMEFENKLTLSVYALIVELSSCFGMLMVLLGPNNLVGNIFPYRAK
>SpOR33
MVQKKKTLRLILNRYQLWNRVLFKGEQPIDTSMDFSLVVLRAIGFWPSKFSEETLTYTIGNSILMFMLTFFILTITA
DLYEASSDIIMFGEDLCVLVGLYLIFLKIILYRIYKSNIQRIVEEFHCLLKKFAFGSTAIRKIQRQSYIRDIVIFNTFVVLG
NFLFVALAFQPIFSGEPRTLPFRAKYPFSIDDQLRFQIVYGFQCFCTLVCIKIIVYIDNVGCLIINQTAANLEIICEKIRE
FRGKKMSREDLGAIIVDHQRIIKLIDYVNKYYYYNFVGQVGASTFLICLTAFEAQLAKDQPLVALKFQVFMFSSFM
QLFYWCWVGNQVRFNSIEVGVAAYETEWITQSPSFRRDLMFLILRAQKPLSFRAQPLYGFDFEAFSKILSSSYSFF
TMLRTISD
>SpOR34

MAPGVFHFSRLDPLEDSFFRISKWILGLLQCWPLDRLNRITKIKLVLHLTILLTAALGEIRSSWLLRKSLLEAIDALSP
GVTKLVTWIKLVCFLIYRKDLELILKSLLEHCKTDTKDSRKNYLIRKISYFNNMCCSVLYINAITTSVSFGFKPIVVWA
YQYFVNEKRNTWVDLPYRAALPYEDMTEMNKPMYTFMYCFLAYSGLVTTFAVSGTDGTFLCFCMYISVAYQCL
QEDFKSTFKVHAANDPKRSKILYNDLSALIRRQQKIIEIFNSFNRVYTFMIFIHFVSASILLALLSINL
>SpOR35
MSRPSEKYLKLMKVLRLCGAACGADVADPNYRVNFITICVIICITVYFIFSVYTVQLKFSENWGVLLESFCMVGSV
FQGIAKLLSGIVYTRHLSDINIEIFTIYEEYEAKTNYYIKVLNNSTARVKFLLKTLSILHVIIFFWLFTAPVIMYLMNGQ
RYMLMQFYLPGLDVETDFGYFTTIGMQFACLVFGGFGNFAGDLFFIVFNMHVTLFADVLKAKIIEFNEIADDNF
EMMKMDHLLNDIIGWHQKYSLFAKTSNKIFDWITFVQIVTTSLSIVLTLFILLTGDWPGSYSYLALELLMLYVYCGI
GTLVEKTNDKFCDEIYAINWYMLAPPQQKCVLLMLMKSQDPELITVGGVMPLSVSTALQVTKSVYSIMMMILN
FIE
>SpOR36
MSSTFFLTTFSQFLSLGIAIARDPTSTSKPYLVNMQFPYNAQEPWKYALTIMYTGWAAFSVVSITSAEDCLIGTTL
SNCTIRYKMLHEDLSKLYENSLKTLQSKPKSKFYRNNQLHEIFHQKLCTIIMKQQILDRFLQDLRSFLSLPIFVVVLF
GIFLLCTVAFQLQKNGISVDTLEYFFWLLAQCLEFFVIGKFGQSCTDASAAMANAYYSCNWESLLSHGNAVDNV
KLIKNISFLVFRAQKSVHLDGMNFMILSLDSIGSAMSSSVSYFMFLSTMEDISNVKNEN
>SpOR37
MSFPFFMQVLLSALVLCFSGYQTSSISPRENPGQFLAMGDFLVVIGIQMFLPCYYGNQVTIESGKMSEALYNSN
WMDLPENSKKVIRLYMEFLKEPVYVRAGKFFDIGLPTFGKTINNAYSYFALLLQMNN
>SpOR38
LKMVQPSGKYLSMMRTARLCATACGADVTDPNFRINIITVFVMICIVVYFVFTIYTVQLKFSESWGILLESFCMV
GSVLQGVAKLIGGIFYSKILCNTNIELCKIYEDFEGKNESCVKVLNKCLEKIKFLLIFMGILYIIIFGWLFVAPLVMYLF
NGRRYMLMQFYFPLLDLETNFGYFTTISMQAVILAFGGFGNYAGDLLFIINNMHVTLFSDLLKIKVEELNAIADK
LDQRNDA

GR

>SpGR1
MALYVTFVLQHSVLSVAMSLYSCVTRTLEMRIVMLNQVLKNLSNQWENRKIRSISAKSRSLNCLDSFSMYTIVTK
NPCEIIHESMEIHHLICEAASTANKYFTYQLLTIISIAFLIIVFDAYYVLETLLGKSKRESKFKTVEFVTFFSCQMILYLIA
IISIVEGSNRAIKKSEQTGGIVHGLLNKAKSPDVKEKLQQFSMQLMHLKINFTAAGLFNIDRTLYFTISGALTTYLIIL
LQFTSSAPNGSVPCSFGQNGNITAIGNVTIAPNN
>SpGR2
MAFWGTGKISKVTPILNPNQKQFLEDEQRYREKLDVLGKKDGSLSEMYVRKEQITDDPKLLDKHDSFYHTTKSL
LVLFQIMGVMPIHRNPPIDNMPRTGYSWTSKQFFWALFIYSMETVIVVMVLKARVNQFVTNSDKRFDEAIYN
VIFISLLFTHFLLPVASWRHGSQVAIFKNMWTNYQLKFLKVTGTAIVFPNLYPLTWGLCIFSWTLSIGVNLSQYYL
QPDFELWYTVAYYPIIAMLNGFCSLWYINCNAFGTASKALAGFLQETLQREKPAQKLTEYRHLWMDLSHMMQ
QLGRAYSNMYGIYCLVIFFTTIIATYGSLSEIIDHGATYKEVGLFVIVFYCMGLLYIICNEAHHASLRVGLDFQTKLLN
VNLTAVDGATQKEVEMFLVAISKNPPIMNLDGYANINRELITSNVSFMATYLVVLLQFKITEQRGLKVQQG
>SpGR3
MFNGFNKHTTKPKTVFLDVKPSFISPPVRRFSGLQTRKDSIALYGTDTRPSLASVTSLENFQKDVLFSNIGASYFF
MRCLGAMPVTRRSPGNAVFSMASIAFAYSVLFFVVLIIYVGYVADNRIKVVSSLKGPFEEAVIAYLFLVNILPILLIPII
WSETRKVAELLNDWNDFEILYFQISGHSVPLRIRKKALGITIALPLLSVLSVVITHVTMADFHILQVIPYCILDNLTA
MLGAWWYLSCEALSDTANILAERFQKALRHIGPAAMVADYRSLWLRLSKLTRDTGTATCYTFTFLNLYLFFIITLSI

YGLMSQLSEGFGIKDIGLAITAIWSICLLFFICDEAHYASHNVRTNFQKKLLMVELSWMNSDAQTEINMFLRATE
MNPSNINCGGFFDVNRNLFKGLLTTMVTYLVVLLQFQISIPDGKPRGNFTFGVGSLLNGTDVVVSLISH
>SpGR4
MFPQNKFILLCKLFCLAPISNSSNVQRILSAYCLITVICLIVVTAFSFSIDIFGEGFVVSSLATSAVFVSGIVGHITVVLE
SLLKKKEHEYFFAKLYELEYIFRFKLRHRIQVRSDRKHILRKYSKLSVVIIICFGAYITIISTHDYLGYFWLASVSVVVNR
MRLIQACVYVDFINFRFDSMNLLLKQVSNSIVQHNLRIPDIDYEIIDNTERIFVLKTIYTRLNKLVLCLNEAFGWSIV
TLTVYYSLDITCNLYWLFLGFEDILDNFHIYECCVTIFPIALLLFALSHTCCKCEKKGDVARQLVQQILVSSTFDHTNY
LLDAFLLQINNQPIVFHAKHFFNVNAKLLPNILSSMLAFLVILIQFQLTKNEL
>SpGR5
MFSHSKLKLILKILALAPSSQKESTQRVLNSFSLITIISVVLSAIFGFTLDVFGDGYIVSSLATSVVFIGLIVGHLAAVLE
SLVKREKHDEFFEKLYELDDFFRVKVGYTIPVASQSKKLNGKYSIITLVVFVSLLIYIGSILTHSYEGFFWHGSISVIVS
RIRCIQASVYVDLFNLRFRHLNKVLKQLASSLLKPSNQMLDINLCDIDNPKRIIVIKSIYRKLYDLVTYLNDAFGWSI
LTFTVYFCLDFTCNCYWLILAWDDYLPSYHNHECFFTVCPIVLLMTSYCYICSECMRMSDNTKLLVQKMLVTSN
NDHTNDLLNAFLMQINNQPIFIHAKMFFRVNLKLLTSVS
>SpGR6
MIRIEDEEFEEVLSYSLLRNDLQTLHKVGKPDANKLLTLNDAARKREQKRSERAKLKSANGDHVEIHDQFYRDHK
LLLTLFRVLAVMPVLRSEPGRISFSWRSGATMYAILFWCLMTIVVLFIGQERVQILITTKDFDEYIYAILFVIYLIPHF
WIPFVGWGVASQVAKYKTSWGTFQLRFYKITGTSLQFPNLKITIYIISVGCLLCAMLFLWLLSIFLDGYPLWHTIAY
YHIITMLNMNCALWYINSRAIKTASHALAKCFKADVSRECHASIISQYRFLWLNLSELLQSLGNAYARTYSTYCIFL
FVNIAIAVYAAFAEIIDKTNIFSISYKEMGFIVDAIYCSGLLFIFCDCSHNATLVVSKGVQETLLSVDLRNVDAQAQK
EIDLFIIAIEMNPAIVSLKGYVNVNRELLTSSISTITIYLLVLIQFRFTLDRGGEEDY

IR

>SpIR1(67c)
LLYLSSTNLSCNLRTLIFEMQIFCYGLILTNIYVAFLSAFLTTTVQDKQIDTLEELLQSGFKIISTHFEVVAIMHTSGFD
QRYNNLFEVENIDVINEYRKSLNKTYAFVFAEDRAIFFLGQQKY
>SpIR2(31a)
MKWNQGMMLLSLKKNYSLENVLTVRNHAKTSVIVDCSCSECKEVLQISSEGKYFNKTYQWMIWDLKNTGLEE
LIGDLNNIGPNAQITYVNRTQLSQSIEIDIETYSFYDVHSKGRHLNAPLEVVHYADWRDGKLMAYQDVLKLQGIR
HRDQFKQLKLRGATVIDQDNIVNDSQIISILSSSGKEDGVAAFTKYHYVLSTILQERFNFTLSFRNLRGWAGRLRG
GIFRLGFLGVVMRNEADVGASGAFNRINRLAEFDTIHQSWKFDSAFMYRFTSDIDADGMSGNFLAPFAMQV
WLMSAATVLLVMVLWYIVAWLVSKTFNEGERDRDGFLLKAFAAVCQQGLDPIPSGIPSRTIVFTLLLFSLVMYNF
YTSSVVGGLLNSSEQGPKTVAEIVNSPLKLSFEDIGYYKVLFKNPTNALVMRMYKTKVLPERGSDELEVFSHIKEA
IPFLKKGGYAFHCERVDAYPEIARHFDANEICDLRIVSGLLDKGLMNFIVTKNSMYTELFRVMMCRARETGIIMR
TLTIHQPRRPECQSSYTVYPVAVSGVLSAFMLLGVGMLFALIVLIGEFLYYRFNERIKMQAQKYLENFCRNSF
>SpIR3(75q2)
MKPSVGLKVLFLGLILGYGIVCSSEIVEFVQPIKSLLNYLNIPLRVQIMICWNFDVRLEFYRRFSQLDGHNGSRNY
QASISDANLMNFPNGILEEHQYLIIVDLNCNQTQRLLFEVGSKLTHRFKWIVLDSVSQLERDSDELILETFKNLEV
MPSSEVYCLAKIEEYSGTVIKQVYRKSMEQELILELFGFCTNTTSLNVTRSSHISSIRRRNLEGLKLRASLVLTHNDS
TNHLTDGLDRHIDTLSKVNYVLTTQLADFINGDIEFKFVKSWGYMNNESKWSGMIGDLVDNRSDIGASALFFTA
DRIKFIEYIAMPTPTRSKFVFRSPKLSYTDNVFLLPFDTRVWHAVIALIVVTSVALVFSTWSEFKIKQNEGNTDPGV
LHPSALDAFILVFGATCQQGSAVSPRSMTSRIIMFIVFLMLMFLYTSYSANIVALLQSPSRKIQTLNDLLKSRLKLG
ADDTIFNRYYFTHTSDPVRQQIYQQKILNKDGSENFMTLEEGVELLREGLFAFHMEIGVGYKVVSETFREDEKCA

LQEIQYLQVIDPWYAIQKNSSYKELLKIGMMRIHEHGLQERENAILYTKKPKCTGGGGKFITASLVDTKPAILTLLW
GYLFALAAIFFEILVFRQLMRYRNYAMDNMDSANN
>SpIR4(40a)
LKITTDIINQEQNEVMPKNLLERCIWFTVQLFLKQSCSEPYNGYRAKFLMIVFWISATYVLADVYSAQLTSQFARP
AHEQPINNLQKLHRAILREGYRLYVEKDSSSLEMLQNGTEIFRNLYALMKKQTDYEGYLIDSVESGIKLIADGLENK
VVMGGRETLYFNMKQFGFKTFQLSQKLYTRYSAVAVQLGCPFLDSLNEVLIRLFEGGILEKMTNAEYEALSRLVD
VNGLTNKFEQQDGVNSNAAAATTDNNKENKKFKNQSSDYIIQPINLRMLQGAFIALGFGWGLAVGIFLLEFFF
GNQYFSVFKRLNGCFQQLCRRLKLTCQNYWKRYFSN
>SpIR5(56d)
MVSKIVLLVFSLVFQAYAQNDSFIELIKRVYKERDTATCHFINFDNDFLAVFFRSNPPIPVSIWNDASIPSFEFSNAIL
VVQALEEETVHNDTLLERLIENLEQRRDVIVSADSLVKLEEYFSYLSEKKFTRIVGLATDDDRILYGYFPYADNKVQ
RISPHGPLSQPLSDLNGFSFRTTIMHDFPRMFSFKDKKSRLHITGSSANIFISFLRKHNATYQQLSNSRRNSSITM
TDVVKATIANEIDISMNAYSPQPSCGYSYPIKVVTCLMMVPVNGFVDTQEYFIRPFKSWVWIFILIFVFYVTVWK
VSLNKFLQKSPDYWQSFSQTFTALLLISPERCITDKLRLHSQVFFFTFLISNIYVIYFTSFMIVSIPIKQFDTMQDLID
NNVKILTTDFDKNHLTEVKAYSKSFLDLMVPVDRKLYGKALLSLNNASFAYIVGDDKRDFLMNIRASWMIKPVLR
TARETLNFYFICYFLHPHSPFKDILDEFIINCLETGLTYKWDTDLVLEILRHGFKEKIYNRPEYGPQNVSLNLKHFKFA
WTFYVIGLTMAGLVFFGELYIIKFVSAEIQNTDARY
>SpIR6(92a)
MSLAELVTFLVNKYFSNLISVLITYNSSSTSEVSGLFLDSVQASFQNLTQQGHFIGINWIDTDVLAKQEDLRGLLLQ
NADIGIEGYITILPSLLEFLNARLYATENSVLRLKDKYYLFLSVDENENPENVLTNEILKLYPHHLFVSFVKNSSDITL
WTQKFVGASDNLKPIHLDTYLKNGEFTKNAELYPNKFLNMFGRILEVASVTYIPYVVSYSVAEGMGDVDNLDPE
QPKKSVIYTGTEADIVLSFCRLRNCTIRVTPCGNDNWGGVYDNGSSDGLIGSVFRHETEFGIGCYYNWFNDLFE
TSIAIAKSAVPILAPAPSLYPAYMTIILPFNKLIWTMIIISIFVSAIVMHVIMYFNTYMEHREDPTKFHFDHASSYELT
QFQMVAIFFQQSFSQSKLDRFATRFFLSTLLLAGITLENTYSGQLKSLLTIPAVTEPVDTIQKFAKTDWQWASPSG
AWIFSIAFSDIPHEKIMTEKFVEMSYEQLRDASYTGNYGIGLERLHGGIYSFGDYIKDGNLDKLIMTKDDLYYDYS
RGFAIRGWPLMDTFNTHILWCLEHGLYNYWEKKHVYYFLDRKTQGIMFNLASGHLQKFPPIPLTIANISGPLWA
LAIGYGMALIVLISEMFANRSNKFIKHAKITKKSHS
>SpIR7(75d)
MHVLPVISIIVAGEILFTTTSATAAAVSSDLTSGSQPTDEDAQTIYDGSNSKVKEQQLSSWWNIANLPLYVDYFRF
HGVGSVGLVLCPEDVSGRDEIVRPIGRLVRGFLASQMYVNVFRDNMNLDHDVDDDDDDDDVHQFTLNPLSY
KTGIIVNLDGSCAWQTLRAAEQRNLFTTNRFWLLLSAGGSGKDETIPDVFIPPDGEVRLLMLSEGSLLDVYKVD
AGKPMRVRQVGRNITSSRDLVTSLQQFGSAISYRENLEGLEFRTGLVIAFPDMYTDIEDLTMRHIDTITKVNYPLT
RALSGRFNISFNTHQTDNWGWKKPNGSFDGLMGRFQRYELDFSQNAIFMRLDRVAVVHFVVETFRIRAGIMF
RQPPLSAVANIFALPFNYDVWIAVAVFIIFTAIIMSVELAFSPLLGGMDFGDCIVFVWGAICQQGFYVNIPNRSAR
ILVFTTFVTTLFLFTSFSANIVALLQSPSEAIGTLGDLGQSPLEIGVQDTVYNKIFFNESTDPVTRTLFQKKIQPKGEN
VYMRPNMGMERVRTGLFAYQVELQAGYQVISDTYAETEKCGLKELEPFKLPILAIPVRKNFPYKELFRRQIRWQR
EVGLMNREELKWFPQKPKCEGSVGGFVSIGLTECRYAFALFGYGFGLAVSMLFVEILMKKLVGLVGNGGEMSV
KN
>SpIR8(64a)
MSSNKYQKNPHTPGISFLFLAIILHRFVNASWTTLQTEKTNIKPKIASYYTRGLLHQMPSPSPSPVPLPSEQLFDS
KRFVDKKSNNIDVNHLESAAGSPGMLHHLIYELVQYKHIPRVSLLTCQQDNGNLDVNVNVNVDVDMGNGSPT
RRQREQTRTSINIDTLTILRSFFTGKDNRRWYKGHTIDGVNISSSFSSSDWIGHNHHSRSRGSAHRRSADSQPKG
VLIKILRIDLLINSQASKSFANKNNSSDTADELLATFLRSEVLRQFVVLDLSCNIASRKVLQMASNKGLFNSSYHWL
LIEDYTFNRQEDDDDEHKNEDEDGDVRDDSQLLQDILTSNAPDTDTASGTSSGDDKINSDGRDADDYGNGNG

NVSDNKVSISRFSSISSDSDNAEKGDQLKQEKDILLIEKFLEKININΙΝTELILAKRINGGPDSDSKKDYYMLYDVW
NPGLQYGGSLNVTQIGNFSEDNGLQLRKWYRQQTTIIRRMDMMNARIRCMIVITNKNHLDNFEDYLTQQYDI
HLDSMHRFNFALLSYVRDLFNFSLVMSRTPSWGYLKNGKFDGMIGALVQRQADIGGSPIFFRIERAKVIDYTTR
TWIARPCFIFRHPRTVRTDQIVFLQPFSNSVWVLVAIFGGITITLLAALTIVEQKMKEGDSDKSSSSVLEEQKMTT
NTIDVKNTKNQHNSLRKTTKMEVDTEVSPFTQKNTMPLARQRQRGLFRTAAPWLANMDEDTNSNTSTSTST
STNTTIINNALTLRTQRFNIKAGGIRKKEDTPVDYGDGAHVLAQSEIDCIDNGVRVSGPINAVVSLNNHGHIEAG
SLKMGKTLRKYFTRSMKYFCKTDGNGNISNKKRWEIMLESMLFYIGSICQQGLSLSTNLISCRCVIITSLLFSFCIY
QFYSASIVGTLLMEKPKTIKTLRNLIQSSLEIGIEDILYNRDFFLHTKDPDAIQLYASVSLPPDQNSTPPTNQTNRSQ
TLEEQHHHWQKEVPTTSTSNHRNRVQSKTSDASNWHDPEYGVAKIKQGGYAFHVDVATAYKIISDTFTEKEICE
LSEIQLFPPQKMVNIVQKGSPLRKIITYGLRRSTEAGLMDYQRKVWHSPKPRCVKQIRTDDLRVDMQTFTSAVF
VLIFGFAASVLVLALEMVHNNLWQQFAT
>SpIR9(41a)
MISIPNSYISVLINFIVQTYHYDATSLCVVFNENFPFTLHQTEADNYVAIRSLYRPTLNETKNIPFDQLDVLSKFEGK
LVESIEYTHCESFIAFEEEIMIFVGSFMKSVLHSKWRSLKNRFLFVYTEGRFDENSAEFDQPFFQDLTNILLIEISKN
GTVFDLKTTKFVGSRKNHPEELVFLDRYYGETNTFEFSNNLYPDKVRDLQGRELISAVFPYEPFAILRNATEKNHFI
DVKDGNEDPSKSHLYDGTDAQILFQLCRLYNCTIQVDTSEEEEWGFVYENYTADGVLGLVFNRKADIGMMAM
YMWMEVYWHLDMSYPFSRDGVRCLVPVPKRLTSWLLPFEPFQVTLWLGLSGCLFLEFLALMFTRKLGKEDIEE
HSWKSSMEFSFISTLKLLISQGTSYIIKSTSLRIIMFSCYIIDIIITSVYSGGLASILTIPAFGEPPDTKERLVKQNFIWGA
TSYAWIASLANTKDEVMLKIKDLFRIYTVKELVEKSLAGEIGVALERLTWGHFAFYKFIDQEIVDKMKLMTEDLYY
AYVVAFTPRPWALLEKFNLCILLIQSSGISKHWEWKVVNDHLDEKLQRQIEDSAWANFGDGGAVQLGLTNFAG
IIVLWIAGIIFSVLVFAVEIISNRKLKPNENLTI
>SpIR10(8a)
MERTKAFICILCCIFLQLFVVKGKDIYLVFLVEPVQNDIINDISTALKDVQQFHYDAEIKENILVNSGNTKSDIENLCS
VLSEHIAFVIDFSYQPWIESTEFYKNIPYIRVDLIISPILKIFANFLKEKSANDVALIFENEQDTEEALFRVIQGYPFRV
VLVNSAENDFIKRLKNLRPCPSYFAVLSRTENMNTIFENINDAKMFARSKEWHFIFLDSKDNDFKYSELVENSTKF
SIKTKEMCEALDIKENYCVSEFKIQKFVVLEIMKRIQEFSANNDLQELRPFSCYNATPYLTEFDLKSEFKESKFLTIN
DNLISYDLNMSISSFSNVPEDITNLALWQNGELKKINATINPAKRFFRIGTAEALPWSYYRRDEQSGEILLDENGQ
PIWEGYCIDFIEILSQYMNFEYELVPPSSGSFGKRFPNGSWDGVVGDLVTGDTDFAVAALKMTSEREEVIDYIAP
YYEQTGISIVIRKAVRQTSLFKFMTVLRLEVWLSIVAALVATAIMIWLLDKYSPYSAKNNKDAYPYPCREFTLKESF
WFALTSFTPQGGGEAPKAISGRILVAAYWLFVVLMLATFTANLAAFLTVERMQTPVQSLDQLARQSRINYTVVE
GSDTHSYFINMKFAEDTLYRLWKELSLNATNDQGRYRIWDYPIKEQYGHILLAINSSIPVKDAAEGFRKVNEHEN
ADFAFIHDSAEIKYQITKNCNLTEVGEVFAEQPYAIAVQQGSHLSDEISYAILELQRDRYFEGLSAKYWNNSLNKE
CSRSEDQEGITLESLGGVFIATLFGLALAMITLAAEVVYYKKKTKAEKSKIIAVKQVKPINSVAGSTPPPTFEAATFR
GRKIPDKITLGDEFKPSITKKKEKLGYFPSQSKDFTPKYYE
>SpIR11(92a.2)
MDSHGRTSLAAGESFGFLTDNQKTKNPIIPQKSKLLFSTISYFLDFRFFFFFWIYSVFLLRTVYPHHLLVSARKCPDL
ELWTQKYVGIEDNLDAVYLDTYYSENRTFAIRNVDFYPNKLVNMLGRTMAVASITYLPYVISYFGTENNGDVDCI
DAALPTKSITYSGCEANIVLTFCELRNCTVKVMPFGKDNWGGVYENGSSDGMIGYVYRQEAELGIGCYYNWY
NDIFETSIVVARSAVTILGPAPSLYPPYMTNILPFGKLIWSLIILSIIVSSVVMHVIKVFSFKMKYKHLERQPEYDHSS
SYILTLFQMVAIFFQQSFSQSKLDRFAARFFLSTLLLAGITLENTYSGQLKSLLTIPLFTDPVDTIKKFSATTWKWGA
PAPAWVLTISDSDIPYERIMAKKFEVRNYGELKNATFSGSYGFGVERLHGGEFTYGDYIEEGALNRMILTKDDLFF
DWTRAFAVRGWPLMDEFNTHILWCFEHGLYKNWERKYINQYYDETTQEKFRFLDAGHGQKFPPATLSIANISG
PLIALVLGYITAFLVFCLEIF
>SpIR12(76b)

MTSGFDLILSAILCFGCNATDPSPQGQTTAVVISVNGTDVAAVSPDLNNNGNFEIYRTKRENMEKMRQYVNGN
HFRIATIEDYPLSYTQKKADGTVVGAGVSFELIDFLAEKFNFTYEVVVPQGNIIGSTGDYKNSLVELLYDGKADLAA
AFLPLLADVRIKCDYSTTVLDEGEWIMVMQRPKESATGSGLLAPFEFWVWILIFISLLAVGPIIYVLIIIRNKITGDD
SQQPYSLGHCAWFVYGALMKQGSTLSPIADSTRLLFATWWIFITILTSFYTANLTAFLTLSKFTLPFNTVNDILAKQ
KHFVTPRGGGIEYSIKNTNESLSILSNMVLNNRATFTENENDTVTLTTFVEKYGYVFVRDRPAINHLLYLDYRYRKL
ISADNEKIHCPFAMAKEPFLKKKRAFAYPFQSNLSTLFDPELLNLVESGIIKHLLAKGLPNAEICPQDLGGTERQLQ
NGDLMMTYYIMIAGFVTSIVVFTTEMLFRFINQRQVGKNNMIIGANELQRSSNFRLHGWAAGRTNHRESLGN
RLNEGIILSNVTPPPAYASIFKHNKNAVVDMNKWKQAVSKNKDESKGVRRLINGRDYMVYKNTNGINQLVPVR
APSATLFQYSYTE
>SpIR13(25a)
MQIRRGFIALLAYIWISTLVNGQTTQNINVLFINEVDNEPATKAVEVVQTFLKKNPNYGLSVQVEQIETNRSDAKI
LLEAICSKYAESIEKKQSPHVIFDTTKSGVASETVKSFSQALGLPTVTSSYGQEGDLRQWRNIDEKKSKYLLQVMP
PADIIPEVIRSIVEYMNITNAAILYDDTFIMDHKYKSLLQNIQARHVITAIAKEGEREQQIEKLRNLDINNFFILGSLQ
SIKMVLESVKPEYFERNFAWHAITQSEGEVRSQRDNATIMFLKPVAYQKNRDRLGSLSTTYNLKQEPQIASAFYF
DLALRTFLAVKEMLQSGAWPKDMEYLTCDDFQGANTPERIIDLRQYFLQVSEATSYGVFELVTQPNLPFNGYSY
MQFEMDISVLQIRGGNSVNERSIGSWVAGLKSSLNVKDEKIMKDLTADTVYRVFTVVQAPFIMKDPTAPKGYK
GYCIDLIDAIAEIVKFDYTIEEVEDEKFGNMDEKGEWNGVVKKLMEKKADIGLGSMSVMAERELVIDFTVPYYD
LVGITIMMQRPSTPSSLFKFLTVLETNVWLCILAAYFFTSFLMWVFDRWSPYSYQNNREKYKDDDEKREFNLKE
CLWFCMTSLTPQGGGEAPKNLSGRLVAATWWLFGFIIIASYTANLAAFLTVSRLDTPVESLDDLAKQYKILYAPLN
GTSAMTYFQRMADIEQMFYEIWKDMSLNDSLTPLERSKLAVWDYPVSDKYTKMWQAMQEAALPISLEEAVA
RVRNSTAATGFAFLGDATDIRYLVMTNCDLQVVGEEFSRKPYAIAVQQGSHLKDQFNNAILTLLNKRQLEKLKEK
WWKNDEIQAKCEKPEDQSDGISIQNIGGVFIVIFVGIGMACITLVFEYWWYKYRKNPRIIDVTEVNVSGKEGGV
DGKNSEGGIILGQSGKEYPYGKSNVALRPRFNQYPNTFKARF
>SpIR14(21a)
MIAKQMNFSFDIIEPKEHAKLGSGESIIEEVRLQRADLGMAGVFLTQERLEKIDMSHGHSRDCAAFITLASKALP
KYRAILGPFQWPVWLALTCIYLGGIVPIVFTDQLTLRHLIGNWGEIENMFWYVFGMFTNSFTFSGKYSWSNSKR
VSTRLLIGFYWLFTIIITSCYTGSIIAFVTLPAFPATVDSVNDLLGLFFRVGTLDRGGWEKWFQNSTHESTAKLYKK
MEFVSTLEEGIGNVTQSFFWNYAFLGSRAQLEYLVQSNFSDDVLGRRSALHLSEECFAPFHIGFVLPKNSVYAEKL
DLAILIAQESGLIAKITNEVSWVMQRSASGKLLQASSSHALKERIQEERQLTTADTEGMFLLMAIGYVLGGLALG
SEIIGGFTNKCRQILRRARKSVSSGISSARNSSDFTTKKDLADYLRRKAIRHESREAAAAADNSGNTFFGFKEFKLT
KATLKELYGTYNQPEPNFIFQNGKLILETDARSIESSARTITTPSSVGEFPLQQEVEEVVQHLDACIDVHDRQQQV
TNSFVLDDIDEKVSEEVENPFGSLVDDVNLVSRYENLKLFEENEHQK
>SpIR15(94e)
LNDNCLRGALGQSFSEMRNAPLKIKFIYLQICLLGIILTTSYNSYLQTYVTSPPTIAKIRSFDDLLESNIKVFCLEEEFQ
ELHKIDHRFRDKYFKIFSFEKDFARYVIFRDTLNTNFAYMVNRVKSITIQEQQKIFTKPLFRHPSELCFFDFIPISFPI
QEDSIFRDAIDFLILQVHSSGLLQFWHKKSFNELIDSGRIKLEDLATKVDFLPMKVDDLRLIWIGLGGGLFVSSLCF
LGEILIDKWKD
>SpIR16(84a)
MAFRIAAFFLFHSIFFGAYFCIQVKSFGDFLEYNHLKQGIIIMCNDDSLQMEELKAISKKSLWLSYIRVGQTTDTN
MNNADFKDLFRQGGGRKVGVSFDIECQSATVTVPILESVSANNFFNSSQAWFILAKSFGMGLQALEKLEINIDA
DITLGIKIQKEDILRLYDLYKVCHQCGTSFESLEKGSWSDSGGMQVLPYFWQTTVQRRRNFSDITLNGGVVINAA
PKGIPLSVYIESYKYKHLDTMQRKTYHLLKIMKEIYNFRIKIVISDLWGVRQNGNWTGIIGKILNGEVAITLSPLRFT
QQRVLVVRYLPEVHVEIARFLFRHPRRSPIRNKFLEPFTPVVWYTVFAITIAGVFLMAITMHHEFENYESVNGSS
AGAGNGAENKLDFFTLIILEAIFLQGPSQMFQFVSTRTLIISICIFAVMLNQFYSAFIVGSLLADVPRTIVSMPALFN

SSLDVGMENIAYNYELFINTSNQMAKDIYVHRIMRNPQTNILPLQKGLERIARGGYAFHVTLVRAYHILKDKLTE
QEFCDLQEVAMEQPFPTCAAVPQGSPYKDHFASSILQLRETGLMAYHDKLWAVDKPQCSAEMMEKDTKVDM
EHFVPALGLLSVAMLTSWMILIVENLVHRQKKIKISFSFKQRQ
